# Spinosaur taxonomy and evolution of craniodental features: Evidence from Brazil

**DOI:** 10.1371/journal.pone.0187070

**Published:** 2017-11-06

**Authors:** Marcos A. F. Sales, Cesar L. Schultz

**Affiliations:** 1 Programa de Pós-Graduação em Geociências, Instituto de Geociências, Universidade Federal do Rio Grande do Sul (UFRGS), Porto Alegre, Rio Grande do Sul, Brazil; 2 Instituto Federal de Educação, Ciência e Tecnologia do Ceará (IFCE), Fortaleza, Ceará, Brazil; 3 Departamento de Paleontologia e Estratigrafia, Instituto de Geociências, Universidade Federal do Rio Grande do Sul (UFRGS), Porto Alegre, Rio Grande do Sul, Brazil; State Museum of Natural History, GERMANY

## Abstract

Fossil sites from Brazil have yielded specimens of spinosaurid theropods, among which the most informative include the cranial remains of *Irritator*, *Angaturama*, and *Oxalaia*. In this work some of their craniodental features are reinterpreted, providing new data for taxonomic and evolutionary issues concerning this particular clade of dinosaurs. The mesial-most tooth of the left maxilla of the holotype of *Irritator* is regarded as representing the third tooth position, which is also preserved in the holotype of *Angaturama*. Thus, both specimens cannot belong to the same individual, contrary to previous assumptions, although they could have been the same taxon. In addition, the position of the external nares of *Irritator* is more comparable to those of *Baryonyx* and *Suchomimus* instead of other spinosaurine spinosaurids. In fact, with regards to some craniodental features, Brazilian taxa represent intermediate conditions between Baryonychinae and Spinosaurinae. Such a scenario is corroborated by our cladistic results, which also leave open the possibility of the former subfamily being non-monophyletic. Furthermore, the differences between spinosaurids regarding the position and size of the external nares might be related to distinct feeding habits and degrees of reliance on olfaction. Other issues concerning the evolution and taxonomy of Spinosauridae require descriptions of additional material for their clarification.

## Introduction

Spinosauridae are among the most iconic dinosaur groups of all time [1]. This status is strengthened every time a new study on them is published (e.g., [2–7]). The history of the knowledge on spinosaurids began in 1912, when the fossil collector Richard Markgraf unearthed a partial skeleton with neural spines up to 165 cm in height from the Bahariya Oasis, western Egypt [8,9]. These remains, which represented the holotype of *Spinosaurus aegyptiacus* Stromer, 1915 and were housed at the Paläontologische Staatssammlung München, were destroyed by a British air raid on Munich during World War II, along with other specimens also referred to that genus [1,8–11].

Consequently, for a long time spinosaurid material was restricted to isolated bones and teeth, although many records were probably misinterpreted as crocodylomorph remains (even prior to the discovery of *Spinosaurus*) [12–14]. However, since the 1980s, new remains have been reported from various parts of the world, providing insights on the anatomy of these theropods (e.g., [1,2,4,5,7,11,15–18]). These records extended the geographic range of Spinosauridae to Europe, Asia, and Gondwanan regions other than Africa, such as South America and perhaps Australia, and its temporal distribution back into the Jurassic [2,16,19–23]. Some authors have also suggested their possible presence in the Late Jurassic of North America [16,24,25].

Spinosaurid remains from Brazil have played an important role in discussions regarding this clade. Firstly, they comprised the first unequivocal record out of Africa and Europe [19,26], despite putative reports from Asia by that time [27]. Secondly, the holotype of *Irritator challengeri* Martill et al., 1996, from the Romualdo Formation (Albian, Araripe Basin), is the spinosaurid specimen with the most complete preserved skull [15]. On the other hand, the holotype of *Oxalaia quilombensis* Kellner et al., 2011, from the Alcântara Formation (Cenomanian, São Luís-Grajaú Basin), represents the largest theropod hitherto known from Brazil, likely reaching c. 12–14 m in length [28]. Despite being smaller than the largest spinosaurid skull reported [5], it provides additional evidence for the wide distribution of large-bodied spinosaurs during the mid-Cretaceous [1,29]. Finally, the Brazilian spinosaurid record also includes isolated teeth and postcranial remains [30–37]. Among them, the most outstanding specimen is a partial postcranial skeleton firstly mentioned in scientific meeting abstracts [38–40] and later described in an unpublished Master’s thesis [41].

Current research on spinosaurids is largely focused on African material, whereas most Brazilian specimens have already been described and redescribed. However, the re-examination of the latter revealed the need for further considerations on a set of their traits. This is particularly true for cranial remains, which overlap other relevant specimens from overseas fossil sites with respect to the known portions of the skull. Moreover, many diagnostic features both at or below family rank for spinosaurids reside in the skull [1,4,5,29]. Here, we provide a reappraisal of spinosaurid skull materials from Brazil, focusing mainly on attributes that were either overlooked or previously interpreted. The new data also provide taxonomic and evolutionary implications for Spinosauridae as a whole, regarding craniodental features and phylogenetic ingroup relationships.

## Geological settings

**Fig 1:**
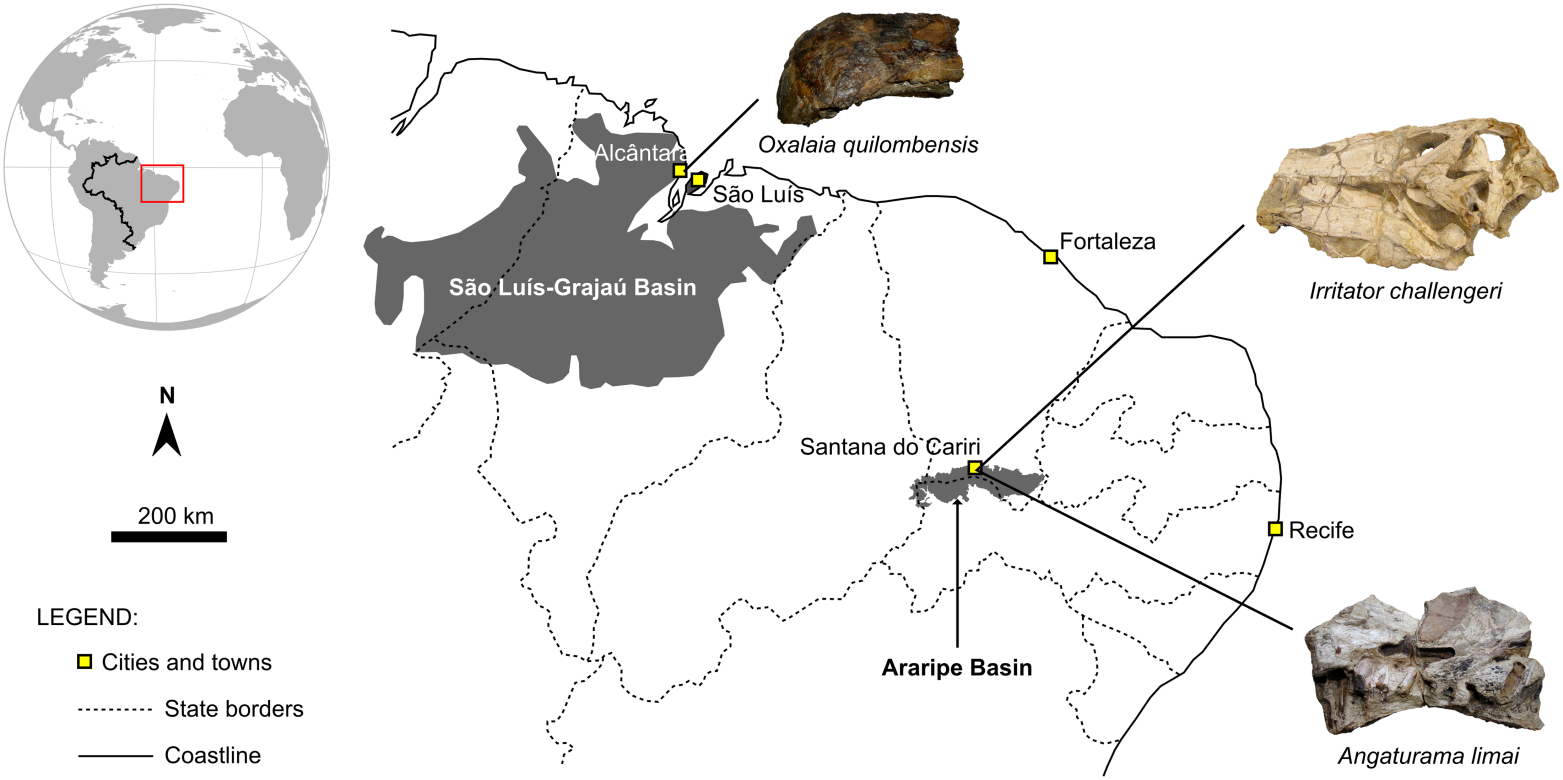
Map of northeastern Brazil showing the location of the Araripe and São Luís-Grajaú basins. The (likely) provenance of the holotypes of local spinosaurid taxa is indicated. Modified from Bittencourt and Langer [51].

Brazilian spinosaurids are Cretaceous in age and were found in the northeastern region of Brazil (Fig 1). Cranial remains correspond mainly to the holotypes of all formally described species, namely *Irritator challengeri*, *Angaturama limai*, and *Oxalaia quilombensis*. The first two taxa are from the concretion-bearing beds of the Araripe Basin, one of the most famous Fossil Lagerstätten in the world [42,43]. For nomenclatural purposes, we will follow here Valença et al. [44], referring to the lithostratigraphic unit of the concretion-bearing beds as the Romualdo Formation. Besides its remarkable and abundant carbonate concretions, usually containing exquisitely preserved fossils, this formation is also characterized by the presence of shales, marls, and limestones. Along with its fossil content, geological data indicate a transitional depositional setting for the majority of the stratigraphic sequence of the Romualdo Formation and a late Early Cretaceous (Albian) age. However, in the western portion of the formation and above the concretion-bearing beds, there are strata containing echinoids and mollusks, thereby evidencing local marine conditions [44–47]. In fact, the holotypes of *Irritator* and *Angaturama* have been found inside concretions, which is common for most Romualdo Formation fossils. Again, as many Romualdo fossils, both spinosaurs lack accurate geographic and stratigraphic information [43,48]. The concretion containing *Irritator* was briefly described as including the ostracod *Pattersoncypris* and scales of the ichthyodectiform fish *Cladocyclus*. It may have come from Buxexé, a locality near Santana do Cariri Municipality, Ceará State [15,49]. On the other hand, the concretion enclosing *Angaturama* was considered as typically resembling those from the Romualdo Formation (Romualdo Member of the Santana Formation in Kellner and Campos [19]). Both cranial remains are distinct yet complementary to each other. Due to coming from the same stratigraphic unit, some authors suggested that they could have pertained to the same specimen (e.g., [4,50]).

On the other hand, the holotypic premaxillae of *Oxalaia*, along with a fragmentary left maxilla referred to the same taxon, came from a fossil site called Laje do Coringa, in Cajual Island, Alcântara Municipality, Maranhão State (Fig 1) [28]. This locality corresponds to the most important fossiliferous outcrop of the Alcântara Formation, which is characterized by sandstones and mudstones intercalated with conglomeratic beds where most fossil remains are found. The majority of these fossils are released from the matrix by the continuous action of tides and waves. Due to its macrofossil and palynological content, the Alcântara Formation is usually regarded as the Cenomanian strata of the São Luís-Grajaú Basin, deposited in a transitional setting related to the opening of the equatorial portion of the South Atlantic Ocean [52–54]. Fossil remains are frequently fragmentary and show evidence of reworking; therefore, some degree of time-averaging is expected for the Alcântara fossil assemblage, which is composed of continental, coastal, and marine taxa. Despite the time-averaging, most of the identified taxa might have been coeval throughout part of the Cenomanian and ecologically related to each other [30,31,54,55]. The holotype of *Oxalaia* was reportedly found in situ, while the referred maxilla was collected as “surface float” [28].

## Materials and methods

### Descriptive basis

As already stated, this work focuses on craniodental features either overlooked or interpreted previously in a different manner. We follow Carrano et al. [29] and Hendrickx and Mateus [56] wherever possible when referring to cranial structures. Regarding teeth and alveoli, we adopt the terminology proposed by Smith and Dodson [57], which was further developed by Hendrickx et al. [58].

Below, we provide a revised diagnosis for each Brazilian taxon in light of the current knowledge on spinosaurids. The revised diagnoses are differential in essence and do not focus solely on autapomorphies, given that distinct features are, in general, independently distributed among all spinosaurids. Therefore, they constitute a unique set of characters for each taxon. However, wherever possible, autapomorphies are temporarily recognized with the caution of each of them being possibly later figured out to be an intraspecific or ontogenetic variation, or even a pathologic condition. This possibility is real as most taxa are represented only by a single individual with the skull preserved, considering formally published specimens [1].

### Cladistic analyses

The cladistic analyses presented here are based on the data matrix of Carrano et al. [29], which was designed for evaluating ingroup relationships of Tetanurae. However, some significant changes were performed. First of all, we incorporated all the changes made by Evers et al. [17], regarding the vertebral character coding of *Carcharodontosaurus*. We then added three character statements and modified others as the result of our new perspective on relative spinosaurid cranial features (see S1 File).

With respect to operational taxonomic units (OTUs), we also performed additional modifications. For example, we added *Oxalaia* as a new OTU in order to investigate the phylogenetic relationships of all Brazilian taxa. In addition, we excluded the OTUs previously regarded by Carrano et al. [29] as wild cards, *Megaraptor* (whose coding did not include recent data [59]), and the spinosaurids whose holotypes correspond solely to non-cranial remains (i.e., *Ichthyovenator* and *Sigilmassasaurus* [16,17,60,61]). *Spinosaurus* is another spinosaurid whose holotype comprises mainly postcranial bones, but normally this taxon has been coded (e.g., [4,29]) considering also cranial remains later assigned to it [5,60,62]. Nevertheless, we consider their attribution as tentative because some of those remains were found either isolated or lacking accurate field data and they only just overlap the holotypic remains described by Stromer [8]. Furthermore, keeping in mind that many mid-Cretaceous African sites are only roughly coeval and the possible evidence indicating the presence of more than one spinosaurid morphotype (and taxon?) therein [17,18,63], it is difficult to accurately attribute isolated skulls to any of the taxa recognized locally. This is the case of the Kem Kem Beds of Morocco, from which comes material also assigned to *Sigilmassasaurus* [17,60,61]. As *Spinosaurus* and *Sigilmassasaurus* may not be synonymous (see Ibrahim et al. [7,64] for a different opinion), it is not possible yet to assign a huge Moroccan partial skull (MSNM V4047, Museo Civico di Storia Naturale di Milano, Milan, Italy) [5] to any of them. The same is true for another skull found in Algeria and tentatively attributed to *S*. *maroccanus* (MNHN SAM 124, Muséum National d’Histoire Naturelle, Paris, France) [62], which is a *nomen dubium* according to Sereno et al. [4] and Carrano et al. [29]. Thus, we prefer not to refer these two non-Egyptian skulls to *S*. *aegyptiacus*. Recently, a partial skeleton, including cranial bones, was proposed as the neotype of *S*. *aegyptiacus* [7]. However, some authors [17] have challenged its attribution specifically to that species. Considering that we could not handle the proposed neotype, which awaits a thorough description, we opted for not including *Spinosaurus* in our analyses. Instead, we coded MSNM V4047 as a distinct OTU in place of the latter.

Another taxonomic issue pervading the choice of spinosaurid OTUs is the possible synonymy between *Cristatusaurus* and *Suchomimus*, both from the Elrhaz Formation (horizon GAD 5; Aptian-Albian?) of the Tegama Group of Niger [4,28,29,62]. The former taxon was the first described, but its remains and proposed diagnosis are thought to be too general to differentiate it from other serrated-toothed spinosaurids [3,4,29]. Shortly after the description of *Cristatusaurus*, *Suchomimus* was described and the provided diagnosis for this taxon is enough to set it apart from *Baryonyx* [2–4,29]. Despite claims of *Cristatusaurus* as a *nomen dubium*, as well as some similarities between it and *Suchomimus* or even *Baryonyx*, we recognize some differences between them with possible taxonomic significance (see Discussion). However, as specifically addressing this issue is not the main goal of this work, for practical purposes we regard *Cristatusaurus* and *Suchomomimus* as distinct OTUs also coded for this analysis.

We also performed one analysis excluding only *Irritator* and another removing only the other Brazilian taxa. We did so because, due to the large amount of missing data, each OTU from Brazil acted as a wild card within Spinosauridae. This procedure recovered more refined hypothetical phylogenetic relationships for *Irritator* and both *Angaturama* and *Oxalaia* in each analysis, which contributed to the discussions regarding the evolution of the craniodental features considered in this work.

Finally, we performed the analysis techniques using the free software TNT v. 1.5 [65], following the same parameters set by Carrano et al. [29].

### Additional institutional abbreviations

MN, Museu Nacional/Universidade Federal do Rio de Janeiro, Rio de Janeiro, Brazil; MNN, Musée National du Niger, Niamey, Republic of Niger; SMNS, Staatliches Museum für Naturkunde Stuttgart, Stuttgart, Germany; UCPC, University of Chicago Paleontological Collection, Chicago, United States; USP, Universidade de São Paulo, São Paulo, Brazil.

## Results

### Systematic paleontology

Dinosauria Owen, 1842 [66]

Theropoda Marsh, 1881 [67]

Tetanurae Gauthier, 1986 [68]

Spinosauridae Stromer, 1915 [8]

*Irritator challengeri* Martill et al., 1996 [49]

#### Holotype and only known specimen

**Fig 2:**
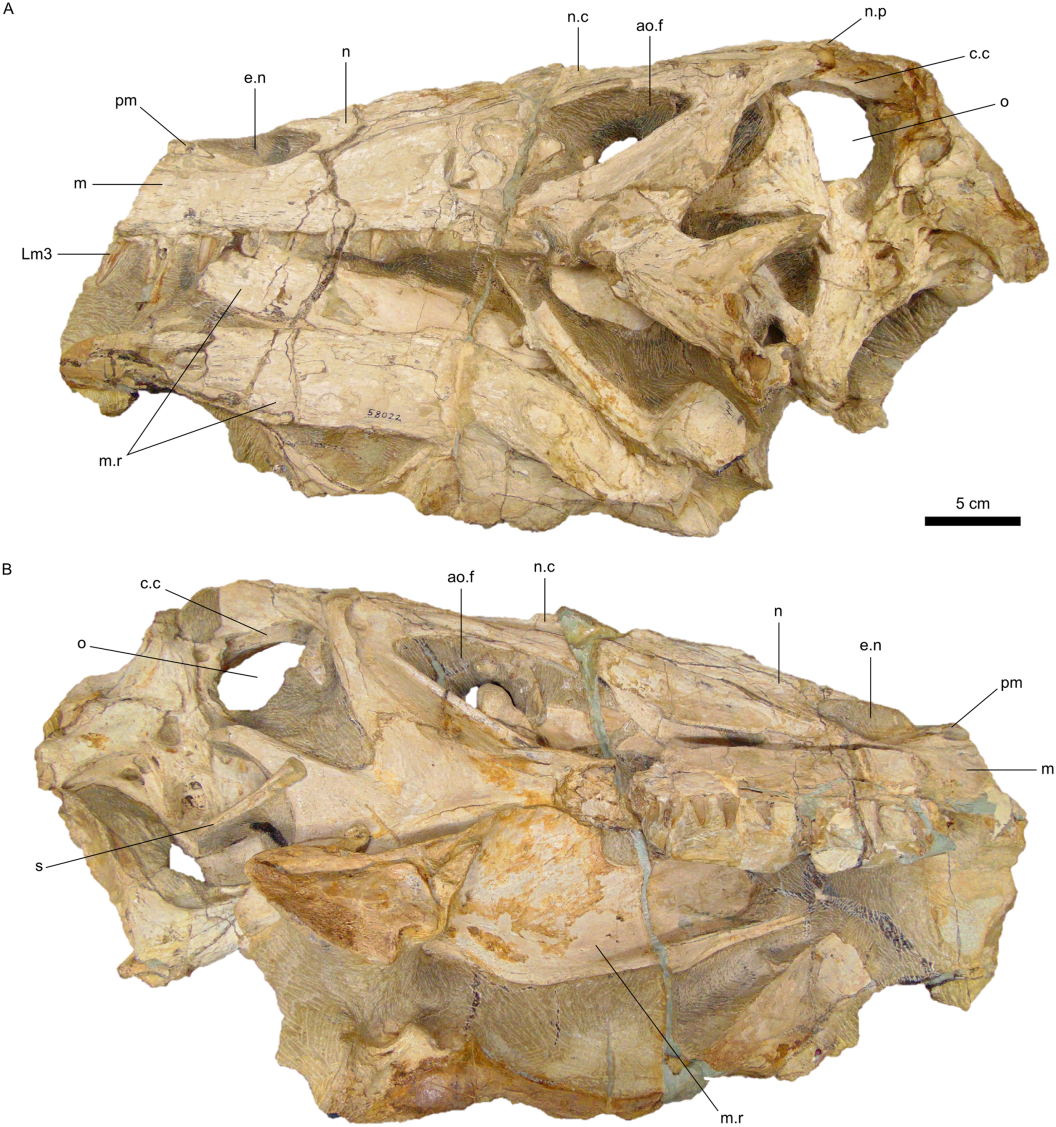
Specimen SMNS 58022, holotype of *Irritator challengeri*. A, Left lateral view. B, Right lateral view. The abbreviation for the third tooth of the left maxilla follows Hendrickx et al. [58]. Additional abbreviations: ao.f, antorbital fenestra; c.c, crista cranii; e.n, external naris; m, maxilla; m.r, mandibular ramus; n, nasal; n.c, nasal sagittal crest; n.p, nasal process; o, orbit; pm, premaxilla; s, stapes.

**Fig 3:**
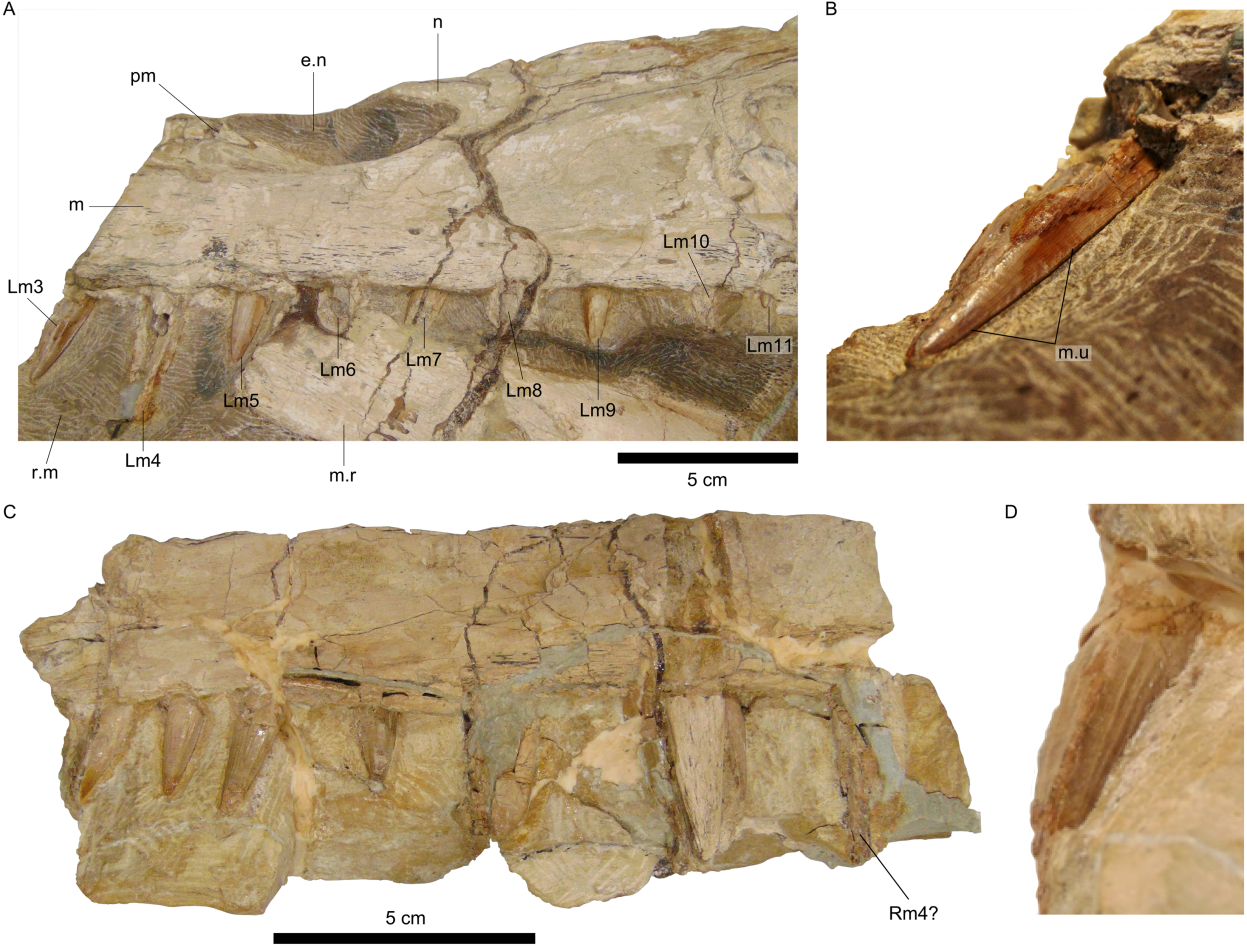
Dentition of *Irritator challengeri*. A, Detail of the left maxillary tooth row. B, Detail of the first tooth crown preserved in situ of the left maxilla. C, Detail of the artificially detached fragment of the right maxilla bearing the right maxillary tooth row. D, Detail of the sixth tooth crown preserved in situ of the right maxilla in mesial view. Abbreviations for teeth follow Hendrickx et al. [58]. Additional abbreviations: e.n, external naris; m, maxilla; m.r, mandibular ramus; m.u, marginal undulations; n, nasal; pm, premaxilla; r.m, rock matrix.

SMNS 58022, an almost complete and articulated skull associated to the mandibular rami, lacking the anterior tip of the rostrum and the dentigerous portions of both mandibulae (Figs 2 and 3).

#### Revised diagnosis

Spinosaurid that differs from *Baryonyx*, *Suchomimus*, and *Cristatusaurus* by the unserrated condition of its teeth and nearly half the number of maxillary alveoli (teeth). Differs from MSNM V4047 and MNHN SAM 124 by the relatively larger and more anteriorly placed external nares and the participation of the premaxillae in the anterior border of the external nares. Finally, an autapomorphy of *Irritator* is the presence of a sagittal crest formed by the conjoined nasals that ends in a knob-like projection over the frontals.

#### Occurrence

Exact geographical provenance unknown, but likely near Buxexé, 5 km south to Santana do Cariri Municipality, Ceará State, Araripe Basin, northeastern Brazil. Romualdo Formation; Albian, Lower Cretaceous.

### Remarks and comparisons

Prior to its assignment to Spinosauridae, the unprepared skull of *Irritator* was thought to be a pterosaur [15]. It was afterwards described as a theropod dinosaur, more specifically as a member of Bullatosauria [49]. Much of this taxonomic confusion was due to the modifications and damages in the specimen, which were likely caused by its collectors or additional personnel that dealt with it before its study. Actually, the etymology of the generic name is an allusion to such problems. After a more careful preparation of SMNS 58022, Sues et al. [15] were able to confirm the spinosaurid identity of *Irritator*, which was firstly proposed by Kellner [26].

Despite these damages, the holotype of *Irritator* is considered to be the most complete spinosaurid skull, at least in relation to the posterior portion (Fig 2) [15,29]. In fact, it is one of the few non-avian theropod specimens with one of its stapes preserved, in this case the right one (Fig 2B). Accordingly, the right side of the skull is best preserved [15]. The amount of data recovered from *Irritator* must have been possible due to its fossilization within an early diagenetic carbonate concretion. This becomes even more reasonable when taking into account the scarcity of (articulated) spinosaurid postnarial bones [2–5,19,62]. In this sense, *Irritator* reveals important aspects of the cranial morphology of Spinosauridae. The skull becomes progressively deeper towards its posterior end so that its dorsal outline projects posterodorsally (Fig 2), resembling the condition inferred for *Baryonyx* [3]. This morphology is followed by other reconstructions of spinosaurid skulls [5,7] but contrasts with that of *Suchomimus* [4].

Although *Irritator* was described and redescribed in detail, some of its craniodental features were not addressed further. For example, this taxon is remarkable because it is the spinosaurid skull with the highest number of in situ preserved teeth. However, due to the missing portion of its rostrum, including the anterior end of both maxillae, neither Martill et al. [49] nor Sues et al. [15] properly identified the preserved maxillary teeth in relation to their position along the tooth row.

The maxillary dentition of *Irritator* is composed of nearly straight conical crowns, which are labiolingually fluted but bear no serration on their carinae (Fig 3) [15]. These features are common in other spinosaurids (e.g., [3–5,8,36]) and, particularly, the absence of serrations is characteristic of Spinosaurinae after Sereno et al. [4]. Some teeth also exhibit short marginal undulations close to the distal carina (Fig 3B) [15]. Regarding tooth size variation, in the left maxilla the first (mesial-most) crown preserved in situ is the second largest, being slightly smaller than the second crown, whereas the seven remaining teeth become gradually smaller (Figs 2A and 3A) [15].

**Fig 4:**
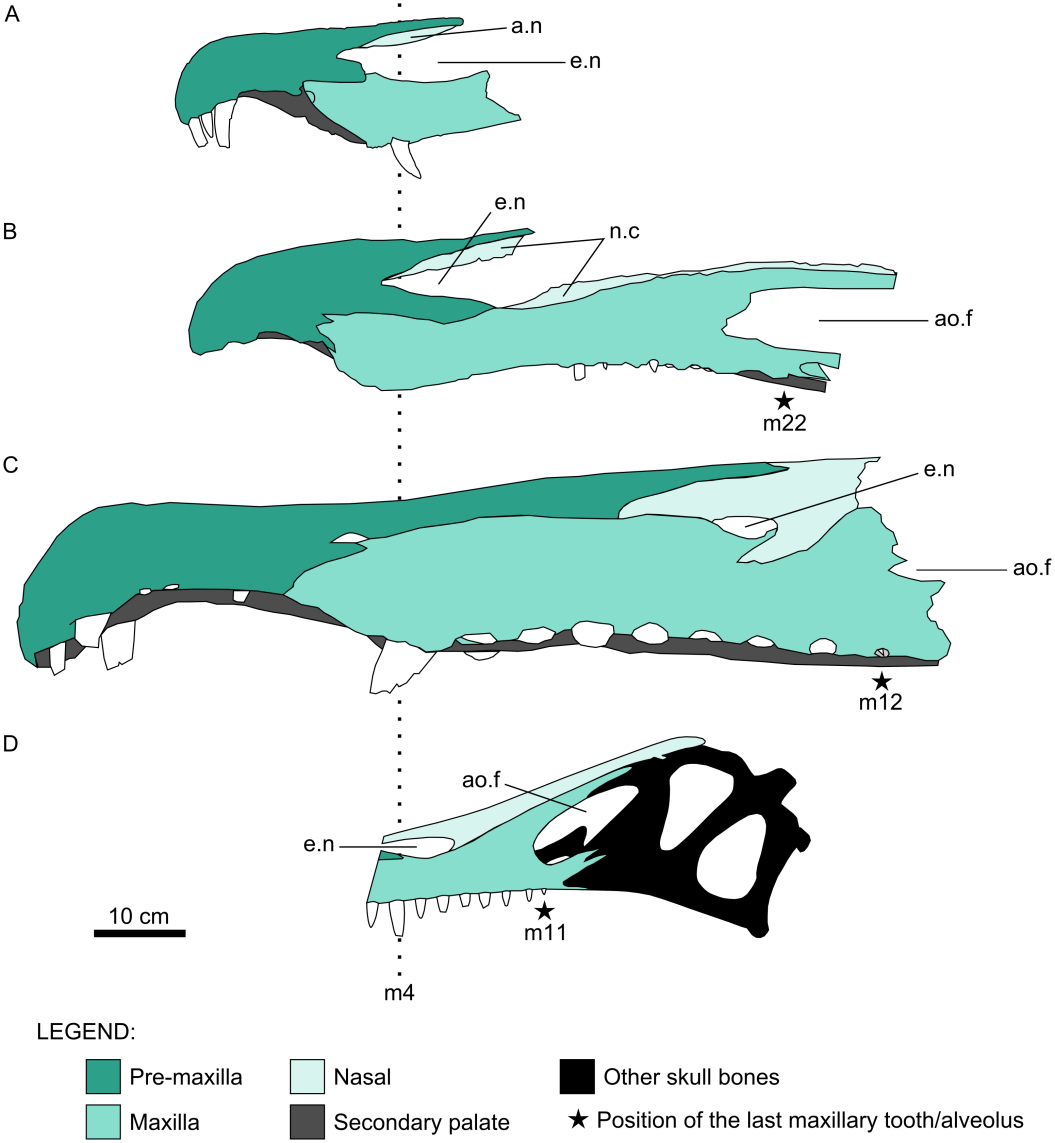
Schematic illustration of spinosaurid cranial remains. A, Premaxillae and maxillae of *Baryonyx walkeri*. B, Partial rostrum (MNN GDF501) referred to *Suchomimus tenerensis*. C, Specimen MSNM V4047. D, Reconstructed skull of *Irritator challengeri*. All specimens are aligned based on the position of the fourth maxillary tooth, whereas the last maxillary tooth (or alveolus) in each is indicated by a black star. Abbreviations for teeth follow Hendrickx et al. [58]. Additional abbreviations: n.c, nasal contact; ao.f, antorbital fenestra; e.n, external naris. A, B, C, and D are modified from Charig and Milner [3], Sereno et al. [4], Dal Sasso et al. [5], and Sues et al. [15], respectively.

In spinosaurid specimens with the maxillary tooth row preserved, the largest crowns (and alveoli) are the third and the fourth. In addition, there is typically an increase in size from m1 to m4, which are followed by progressively smaller teeth. Thus, the spinosaurid maxillary dentition comprises a tooth size variation that begins with a tendency of increasing, reaching its maximum at m4, regarding the crown base length and crown height, and continues with an opposite trend. This is the case both for Baryonychinae, i.e., *Baryonyx* and *Suchomimus*, and Spinosaurinae, such as MNHN SAM 124 and MSNM V4047 [3–5]. This pattern is widely distributed among spinosaurids and no current evidence indicates that this condition is not also present in *Irritator*. The tooth size variation seen in the left maxilla of the Brazilian taxon matches the described pattern, so that the first two preserved teeth correspond to Lm3 and Lm4. This implies a total number of eleven left maxillary teeth (Figs 2A and 3A). Accordingly, Sues et al. [15] inferred that the left maxilla would have comprised at least 11 teeth, although they neither justified their claim nor attributed a specific location within the tooth row for any of the preserved crowns. Furthermore, this number is nearly identical to that of the spinosaurine MSNM V4047, which displays 12 maxillary teeth (Fig 4C and 4D) [5]; this dissimilarity could be explained by the difference in size, interspecific variation, different ontogenetic stages, alveolar remodeling after the loss of teeth in vivo, other dental abnormalities, etc. [28,69].

Regarding the artificially detached fragment of the right maxilla of *Irritator*, nine teeth are preserved in situ as well as the broken base and partial impression in the matrix of the tenth (distal-most) crown. The second and the fourth are replacement teeth (Fig 3B). In general, the first four crowns are more damaged than those from the left side. This precludes an accurate estimation of their dimensions, although the mesial-most crown seems to have been the largest one. If the latter was indeed the largest, the preserved portion of the right maxilla would also comprise teeth from m4 to m11.

The identification of the maxillary teeth of *Irritator* implies the reinterpretation of the location of its external nares. Dal Sasso et al. [5] considered that these were restricted to the mid-part of the maxillary tooth row. However, their anterior-most outline must have been placed somewhere between m3 and m4 (Figs 2A, 3A and 4D). Therefore, the external nares of *Irritator* begin in a comparable position to those of *Baryonyx* and *Suchomimus*, in which the external nares start between m2 and m3 and between m3 and m4, respectively (Fig 4A and 4B) [3,4]. On the other hand, the external nares of MSNM V4047 start only at the level of m9 (Fig 4C) [5].

Another difference between *Irritator* and African material attributed to *Spinosaurus* is the relationship between the bones that bind the external nares. In the former genus, the premaxillae form a minor part of the ventral outline of the nares, whereas their dorsal and ventral outlines are surrounded by the nasals and maxillae (Figs 2A, 3A and 4D). In MSNM V4047, the premaxillae are completely excluded from the external nares (Fig 4C) [5]. Both spinosaurids differ from *Baryonyx* and *Suchomimus*; in these, the premaxillae are major components of the external nares, but they differ from each other in relation to the contribution of the maxillae. In *Baryonyx*, the maxillae form the posterior half of the preserved ventral border of the left naris (Fig 4A) [3]. In *Suchomimus*, the maxillae are minor components of the ventral outline of the nares and are constricted by the premaxillae and descending rami (= maxillary processes) of the nasals (Fig 4B) [4]. Furthermore, Dal Sasso et al. [5] considered the exclusion of premaxillae from the nares of MSNM V4047 as an autapomorphy of *Spinosaurus*. Among spinosaurids, MSNM V4047 is also unique in presenting premaxillae, maxillae, and nasals meeting at a single point, whereas *Irritator*, *Baryonyx*, and *Suchomimus* do not present such a triple joint (Fig 4).

Another important issue related to spinosaurid external nares is their size. The external nares of *Irritator* are both absolutely and comparatively (in relation to the antorbital fenestrae) smaller than those of the baryonychine *Suchomimus* and, possibly, *Baryonyx* (Fig 4A, 4B and 4D). On the other hand, they are larger than those of the spinosaurine MSNM V4047 in absolute length (Fig 4C and 4D), despite the Brazilian taxon being inferred to have had a shorter skull (c. 60 cm) [15] than the African specimen (c. 175 cm) [5,15].

*Irritator* is also unique in displaying a sagittal crest formed by the conjoined nasals that ends in a knob-like projection overhanging the frontals. In fact, where observable, the unfluted condition of the nasal crest of *Irritator* might also distinguish it from an isolated fragmentary pair of fused nasals from Morocco (UCPC-2) [5] and those reported by Ibrahim et al. [7] (and, consequently, the taxon to which they belonged). Finally, additional differences between *Irritator* and African material assigned to *Spinosaurus* might include features of the quadrates, which were well addressed by Hendrickx et al. [18] and, as such, are not further considered here.

### Systematic paleontology

Dinosauria Owen, 1842 [66]

Theropoda Marsh, 1881 [67]

Tetanurae Gauthier, 1986 [68]

Spinosauridae Stromer, 1915 [8]

*Angaturama limai* Kellner and Campos, 1996 [19]

#### Holotype and only known specimen

**Fig 5:**
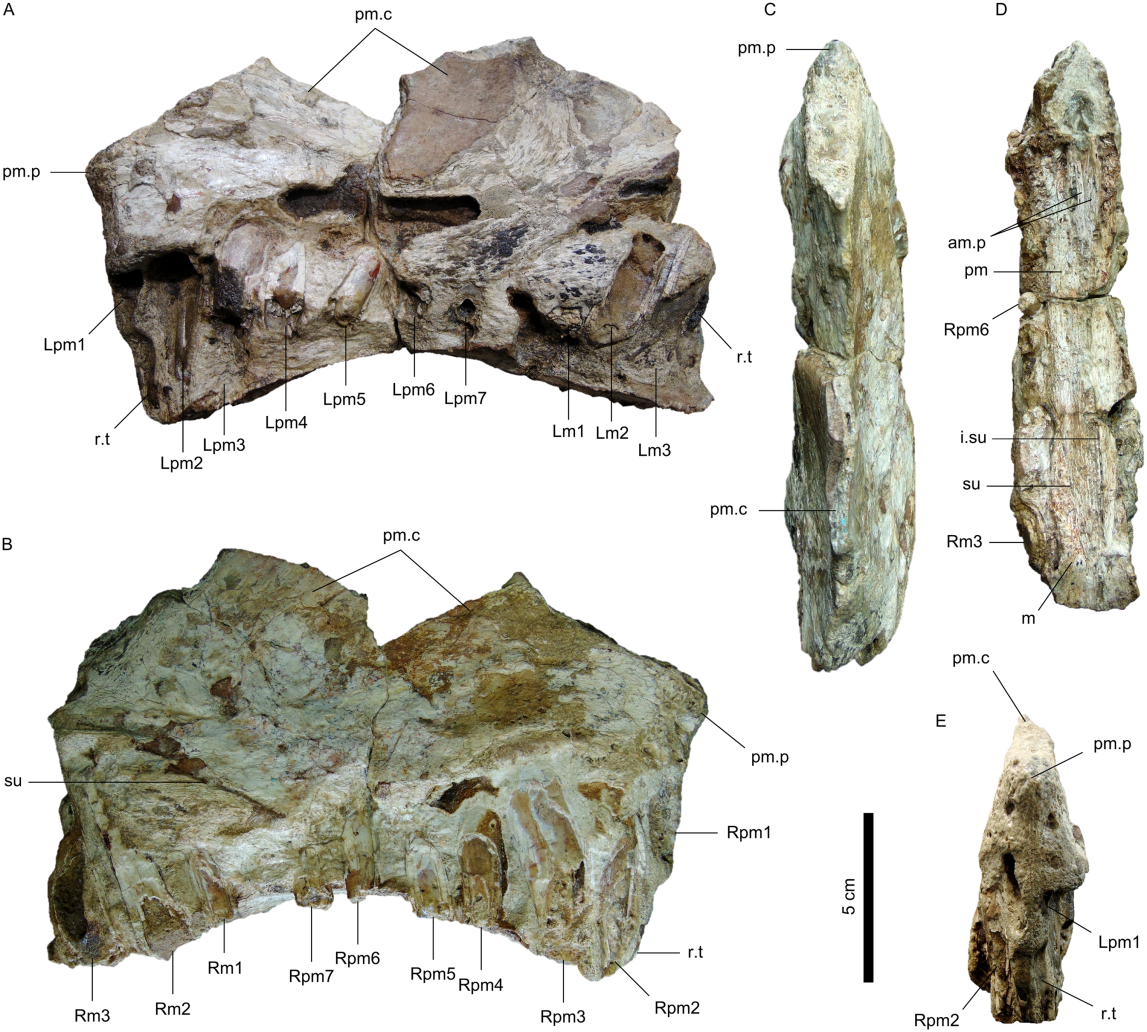
Specimen USP GP/2T-5, holotype of *Angaturama limai*. A, Left lateral view. B, Right lateral view. C, Dorsal view. D, Detail of the secondary palate. E, Anterior view. Abbreviations for teeth follow Hendrickx et al. [58]. Additional abbreviations: am.p, anteromedial process of maxilla; i.su, intermaxillary suture; m, maxilla; pm, premaxilla; pm.c, premaxillary sagittal crest; pm.p, anterior premaxillary projection; r.t, replacement tooth; su, suture between premaxilla and maxilla.

USP GP/2T-5, the tip of a rostrum, comprising both premaxillae and the anteriormost portions of both maxillae (Fig 5).

#### Revised diagnosis

Spinosaurid that differs from *Baryonyx*, *Suchomimus*, and *Cristatusaurus* by both the possession of unserrated teeth and the comparatively smaller first premaxillary tooth. It also differs from all other spinosaurid skulls by the presence of a dorsal sagittal premaxillary crest that nearly reaches the anterior end of the snout, beginning much more anteriorly than in *Baryonyx*, *Suchomimus*, and *Cristatusaurus*. Another autapomorphy of *Angaturama* is the rounded and knob-like anterodorsal projection of the conjoined premaxillae.

#### Occurrence

Unspecified locality in the Araripe Basin, northeastern Brazil. Romualdo Formation; Albian, Lower Cretaceous.

### Remarks and comparisons

*Angaturama* was described shortly after *Irritator*, but it was the first theropod from the Araripe Basin to be considered as a spinosaurid [19,26,34,49]. Given that those taxa were proposed based on supposedly non-overlapping cranial materials (Figs 2 and 5), they could not be compared to each other [15]. Also, for the same reason, *Angaturama* cannot be fully compared to the holotype of *Spinosaurus* [8].

The premaxillae of *Angaturama* were firstly distinguished from other spinosaurids based on the following features: 1) the degree of lateral compression of the rostrum, which is narrowest at the level of teeth pm6; 2) less broad mediolateral width; and, 3) a well-developed dorsal sagittal crest (Fig 5). Among these, only the last feature is still held as an autapomorphy of that genus, but in a modified version [29]. Given that *Baryonyx*, *Cristatusaurus*, and *Suchomimus* also exhibit a dorsal sagittal rim on their premaxillae [3,4,62], this sort of structure is not exclusive of the Brazilian taxon, although it is indeed more conspicuous and, more importantly, extends more anteriorly than in baryonychine spinosaurids [29].

Regarding the mediolateral compression of the rostrum of *Angaturama*, it was initially considered as a natural feature instead of the result of postmortem compression [19]. The expanded condition of the premaxillae, which are often referred together as a “terminal rosette”, appears less broad than in other spinosaurids [15,19]. This might be a consequence of the less pronounced constriction caudal to the rosette, i.e., at the level of the last two premaxillary teeth in *Angaturama* [5]. However, the degree of compression of the holotype might not reflect the original condition, although it may be still regarded as less broad in overall shape. For instance, the ventral borders of both premaxillae are not preserved and some in situ partial teeth are longitudinally sectioned, which consequently reduce the total width of the preserved rosette (Fig 5A and 5B). Additional evidence of postmortem compression is in the palate. As other spinosaurids, *Angaturama* presents a secondary palate formed by the palatal portions of the premaxillae along with the anteromedial processes of the maxillae [4,5,62]. These processes in spinosaurids are positioned between the palatal portions of the premaxillae and were firstly misidentified as belonging to the vomers [3,19]. Thus, the sagittal plane of the spinosaurid rostrum should pass through the medial sutures between the premaxillae and maxillae and equally divide the palate. Nevertheless, contrary to this reasoning, the left half of the palate of *Angaturama* is slightly narrower than the right (Fig 5D) and this might be an indication that the left side underwent some degree of postmortem compression.

Along with the condition of the dorsal sagittal crest, *Angaturama* bears another remarkable feature that differs from all other spinosaurids with the tip of the rostrum preserved. The premaxillae form a protuberance on their anterior outlines close to their dorsal surfaces (Fig 5). This feature implies that the dorsal outline of the premaxillae ends more anteriorly than the ventral one, whereas in other spinosaurid skulls the condition is opposite—the dorsal outline more gradually slopes anteroventrally (Fig 4) [3–5,62]. It is possible that the sagittal crest extended anteriorly and partially over that projection, as the latter is covered by a break indicating that it misses some dorsal portion of the bone (Fig 5C).

One could argue that, as the premaxillae of the holotype lack their ventral portions in lateral view, part of the anterior margin of the premaxillae of *Angaturama* is also missing just below the protuberance. Accordingly, the alveoli of pm1 are incompletely preserved. In this sense, this structure would not be natural. However, in spinosaurid theropods, the first premaxillary alveoli are usually separated from the anterior margins of the premaxillae by a distance smaller than half their diameters. A hypothetical reconstruction of the missed anterior borders of the premaxillae of *Angaturama* would not significantly extend them anteriorly in relation to what is currently preserved. Furthermore, the bone surface of the protuberance and the surrounding region does not present any sign of having been severely damaged, as it would be after losing a significant portion of the bone by taphonomic causes or preparation techniques. Thus, the anterior outline of the premaxillae of *Angaturama* must have been relatively straight or at least concave just below the anterior salience seen in the holotype, which does seem to be a natural feature.

The premaxillae of *Angaturama* present the typical condition of seven premaxillary teeth of spinosaurids. Regarding the size variation within the preserved tooth row, it was described as increasing from pm1 to pm3, gradually decreasing from pm3 to pm6, increasing again from pm6 to m3, and finally decreasing from m3 to at least m4 [19]. Kellner and Campos [19] only figured the right side of *Angaturama*, in which there is no indication of m4, but in the left side there is indeed one partially preserved alveolus after m3. However, considering its smaller size and distolingual position in relation to Lm3, this alveolus must correspond to a replacement tooth (Fig 5A). This inference is in accordance with the aforementioned pattern of tooth size variation of spinosaurids. Besides this replacement tooth and the one associated to Lm2 previously reported by Kellner and Campos [19], there are others incompletely preserved. They can be noticed more clearly in association with Lpm4, Lm1, and Rpm1. The replacement tooth associated to Lpm1 is likely indicated by the empty alveolus between the alveoli of Lpm1 and Lpm2. Finally, with respect to the tooth ornamentation, enamel flutes are seen in the lingual side of Rpm6 [19].

### Systematic paleontology

Dinosauria Owen, 1842 [66]

Theropoda Marsh, 1881 [67]

Tetanurae Gauthier, 1986 [68]

Spinosauridae Stromer, 1915 [8]

*Oxalaia quilombensis* Kellner et al., 2011 [28]

#### Holotype

**Fig 6:**
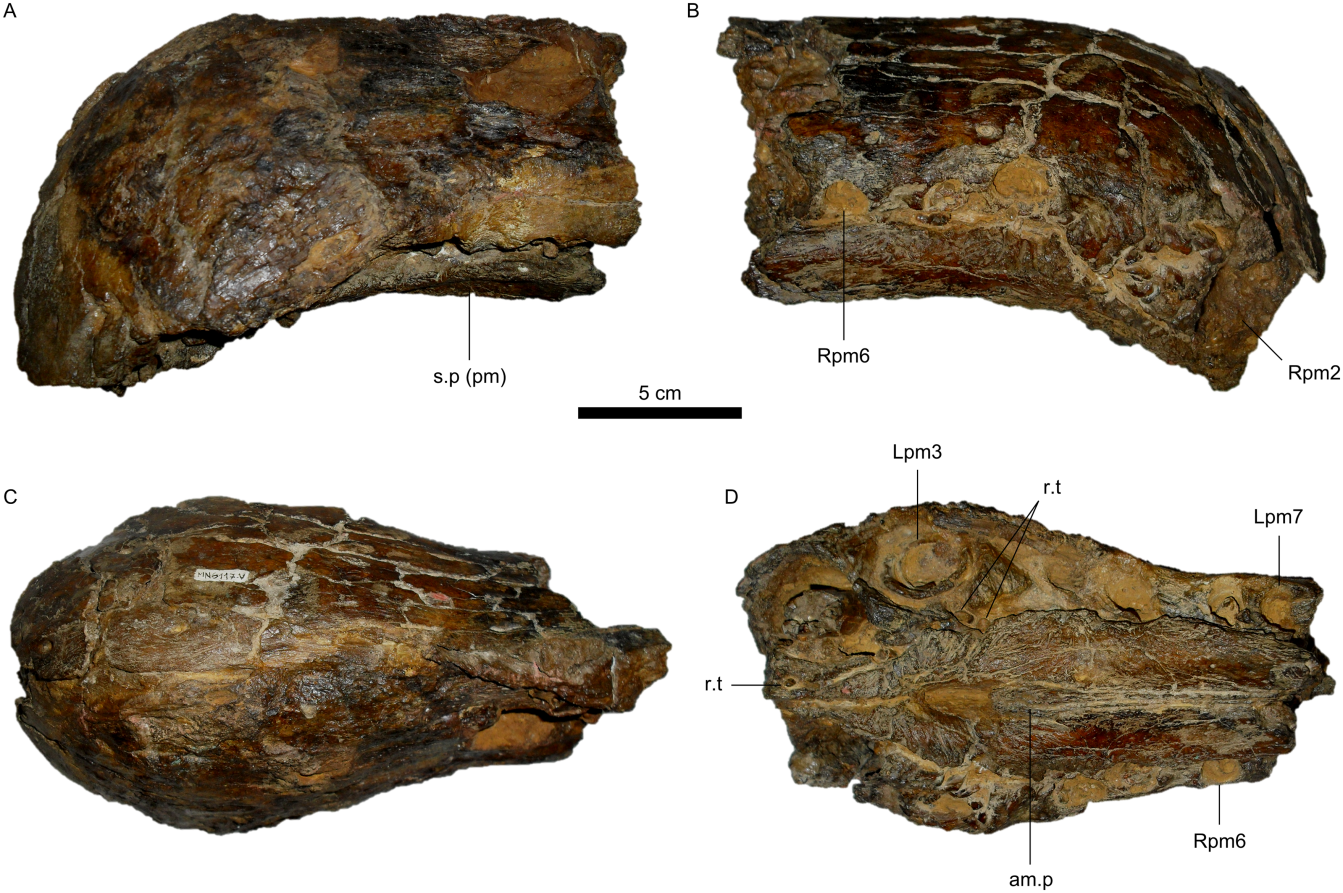
Specimen MN 6117-V, holotype of *Oxalaia quilombensis*. A, Left lateral view. B, Right lateral view. C, Dorsal view. D, Slightly oblique ventral view, emphasizing the sculptured condition of the palatal portion of the left premaxilla. Abbreviations for teeth follow Hendrickx et al. [58]. Additional abbreviations: am.p, anteromedial process of maxilla; pm, premaxilla; r.t, replacement tooth; s.p, secondary palate.

MN 6117-V, fused premaxillae (Fig 6).

#### Referred specimen

MN 6119-V, an isolated fragment of a left maxilla.

#### Revised diagnosis

Spinosaurid that differs from *Baryonyx*, *Suchomimus*, and *Cristatusaurus* by both the possession of unserrated teeth and the relatively much smaller first premaxillary tooth. The lack of a dorsal sagittal premaxillary crest or rim also differs in relation to those taxa and *Angaturama*. It differs from MNHN SAM 124 and MSNM V4047 by oval-shaped conjoined premaxillae in dorsal view due to a weaker lateral compression of the rostrum just posterior to m3, whereas in both African specimens the fused premaxillae are mushroom-shaped. Finally, autapomorphies of *Oxalaia* include the possession of two replacement teeth associated with functional pm3 and the sculptured condition of the palatal portion of the premaxillae.

#### Occurrence

Laje do Coringa locality, Cajual Island, Alcântara Municipality, Maranhão State, São Luís-Grajaú Basin, northeastern Brazil. Alcântara Formation; Cenomanian, Upper Cretaceous.

### Remarks and comparisons

*Oxalaia* is the latest described spinosaurid taxon from Brazil. Similar to *Angaturama*, its holotype comprises only conjoined premaxillae [28] and cannot be compared to *Irritator* or the holotype of *Spinosaurus*. Even the tentatively referred left maxilla of *Oxalaia* cannot be ideally compared to those taxa, given its fragmentary nature.

Despite representing only a minor portion of the skull, the premaxillae of *Oxalaia* are very informative. The small alveoli of teeth pm1, whose diameters are less than half of the following elements, are evidence of closer affinities between *Oxalaia* and spinosaurine spinosaurids, such as *Angaturama*, MSNM V4047, and MNHN SAM 124. Such phylogenetic relationships are also corroborated by the unserrated condition of teeth and the arrangement of premaxillary teeth in those taxa, especially with respect to the presence of broad diastemata [4,5,28,62]. On the other hand, *Baryonyx*, *Suchomimus*, *and Cristatusaurus* have serrated teeth and their premaxillary tooth rows present narrow diastemata [3,4,62] (see Kellner et al. [28] for a different opinion on the diastemata of *Suchomimus* and *Cristatusaurus*).

Within Spinosaurinae, *Oxalaia* resembles the African specimens more than *Angaturama*, as the latter bears a dorsal sagittal premaxillary crest (Fig 6A and 6B). However, *Oxalaia* can be set apart from MSNM V4047 and MNHN SAM 124 based on certain features, such as the aspect of the premaxillae in dorsal view (Fig 6C). In *Oxalaia*, these conjoined bones end in a more rounded tip, whereas MSNM V4047 and MNHN SAM 124 present tips that are more acute. Also, the constriction of the premaxillae posterior to teeth pm3 is much weaker in *Oxalaia* than in the other specimens (Fig 6C and 6D), as previously noticed by Kellner et al. [28]. The combination between a more acute tip and a stronger compression results in mushroom-shaped terminal rosettes in the African material, whereas this structure is more oval (triangular after Kellner et al. [28]) in *Oxalaia*.

Another difference involves the diastemata between pm5 and pm6 of *Oxalaia*, which were considered longer than those in baryonychines but similar to and shorter than those in MNHN SAM 124 and MSNM V4047, respectively [28]. However, this statement deserves some consideration. Firstly, the difference regarding the size of the diastemata might be related to the skull length, as the holotype of *Oxalaia* was likely intermediary in size between baryonychines and MSNM V4047 [3,4,28,70]. This, in turn, might be somehow related to ontogeny. More importantly, these diastemata can differ in size between the left and right premaxillae within a single specimen. For instance, in *Oxalaia*, the diastema between Lpm5 and Lpm6 is slightly longer than that between Rpm5 and Rpm6 (Fig 6D), and the same is true for MSNM V4047 [5]. In fact, the right diastemata of these specimens are comparable in size, despite the left diastema of the latter specimen being longer than that of *Oxalaia*. In addition, the size of the diastema between Lpm5 and Lpm6 of MSNM V4047 may be artificial. Whereas most spinosaurs present seven premaxillary teeth, that specimen presents six [5], which was suggested as a possible autapomorphy of its taxon [28]. The holotype of *Baryonyx* presents six and seven teeth in its left and right premaxillae, respectively [3], and this intraindividual variability seems to be a consequence of the closure of one alveolus, more specifically Lpm7. In MSNM V4047, teeth pm6 are not aligned. Lpm6 is well posterior to Rpm6, but its location coincides with that expected for Lpm7. In this sense, it is possible that Lpm6 is indeed Lpm7 and the true alveolus for Lpm6 was closed at some point in the ontogenetic development of the animal, as in *Baryonyx*. This hypothesis was also considered by Kellner et al. [28] but without specifying exactly which alveolus(i) might have closed. On the other hand, in the right premaxilla of MSNM V4047, the alveolus for Rpm7 could also have been closed, which would have resulted in the misalignment between Rpm6 and the currently observed Lpm6. As such, six premaxillary teeth would not be an autapomorphic feature of MSNM V4047, especially if it is later shown to pertain to the same taxon of MNHN SAM 124, which presents pm7. Thus, the difference in diastema length or number of premaxillary teeth between spinosaurine spinosaurids is not a reliable characteristic for taxonomic purposes.

Nevertheless, the association of two replacement teeth with pm3 could be a unique feature of *Oxalaia* if it is not shown to be an intraspecific variation. The diagnosis of this taxon included additional proposed autapomorphic traits, such as the very sculptured palatal portion of the premaxillae [28]. These structures form a convex secondary palate, which appears ventrally to the border of the premaxillary tooth row in lateral view (Fig 6A and 6B). Other spinosaurid skulls also have this secondary palate. In *Baryonyx* and *Cristatusaurus*, this condition is found in the premaxillae (Fig 4A) [3,62]. It is present in the tooth row as a whole of MNHN SAM 124 and MSNM V4047, although it is also more evident in the premaxillae of these spinosaurine spinosaurids (Fig 4D) [5,62]. This feature cannot be verified in *Angaturama*, as it lacks the ventral borders of the premaxillary tooth row; however, in *Irritator* the secondary palate appears ventrally to the tooth row in anterior view, despite being not evident in left and right lateral views. On the other hand, *Suchomimus* also presents a convex secondary palate, but it does not appear ventrally to the tooth row and, hence, can only be seen through breaks in the lateral walls of the rostrum (Fig 4B) [4].

Another autapomorphy of *Oxalaia* proposed by Kellner et al. [28] is the very thin anteromedial processes of the maxillae, which was thought to contrast to the wider processes of, for example, MNHN SAM 124, although the authors did not specify their degree of thinness. The African skull preserves only the anterior end of the left process [62], which in our opinion does not seem to be significantly wider than that of the Brazilian taxon. Also, the width of these processes varies along its length and, at their posterior half, the processes of *Suchomimus* also seem to be nearly as thin as those of *Oxalaia* [4]. In the described specimens, these processes show varying degrees of anterior extension, which correspond to different widths. This is due to the space between the premaxillae becoming wider anteriorly and being filled by the anteromedial processes. In turn, the degree of anterior extension (or ossification) of these processes might be related to the ontogenetic stage of the specimen, being the greatest in MSNM V4047 [5]. Hence, the thinness of the anteromedial processes might be another character inadequate for diagnoses of spinosaurid theropods.

### Cladistic analyses

**Fig 7:**
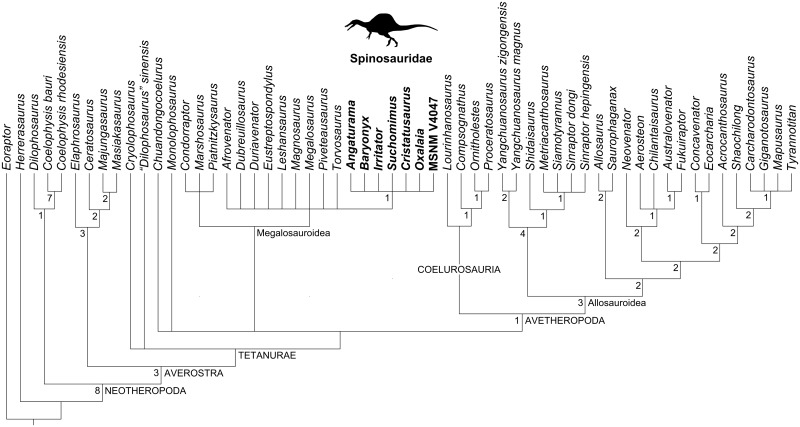
Strict consensus cladogram (1069 steps; CI = 0.409; RI = 0.666) of 145 MPTs obtained from the current cladistic analysis. Spinosaurid taxa are in bold. The spinosaurid silhouette is from Sales et al. [71].

**Fig 8:**
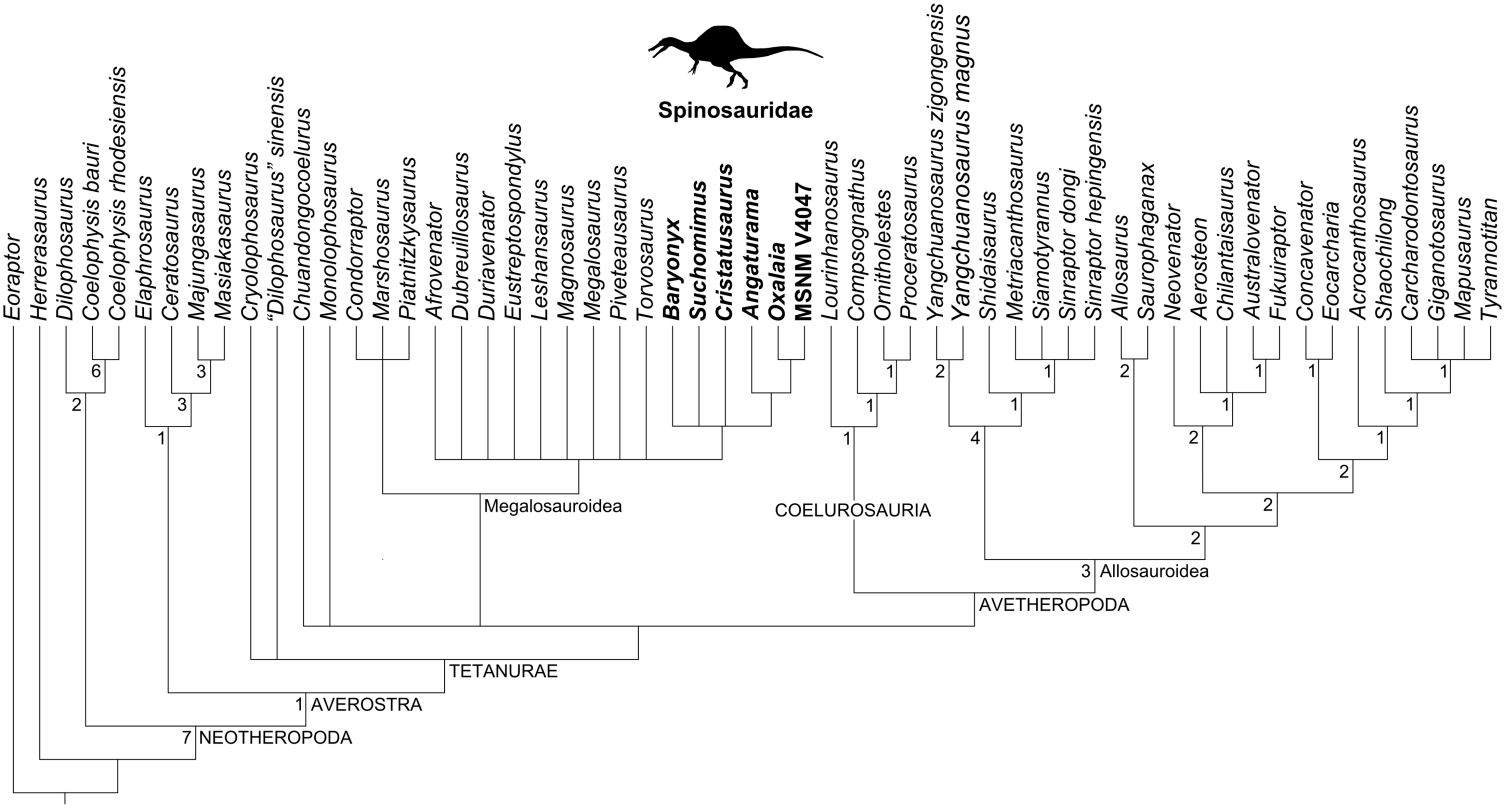
Strict consensus cladogram (1051 steps; CI = 0.415; RI = 0.670) of 150 MPTs obtained from the current cladistic analysis excluding *Irritator*. Spinosaurid taxa are in bold. The spinosaurid silhouette is from Sales et al. [71].

**Fig 9:**
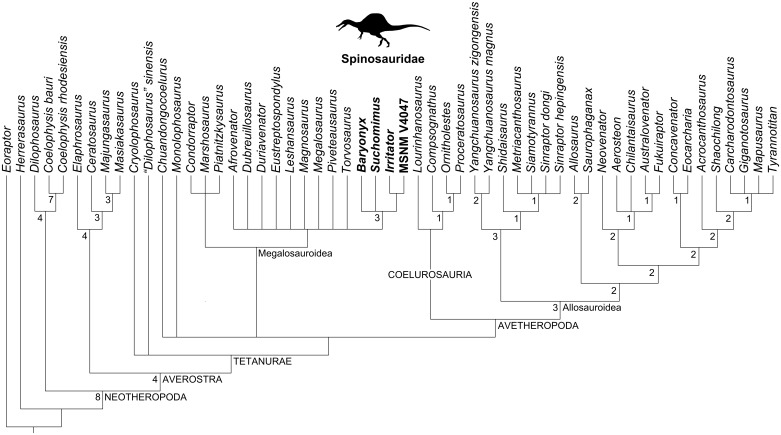
Strict consensus cladogram (1056 steps; CI = 0.413; RI = 0.664) of 148 MPTs obtained from the current cladistic analysis excluding *Angaturama and Oxalaia*. Spinosaurid taxa are in bold. The spinosaurid silhouette is from Sales et al. [71].

**Fig 10:**
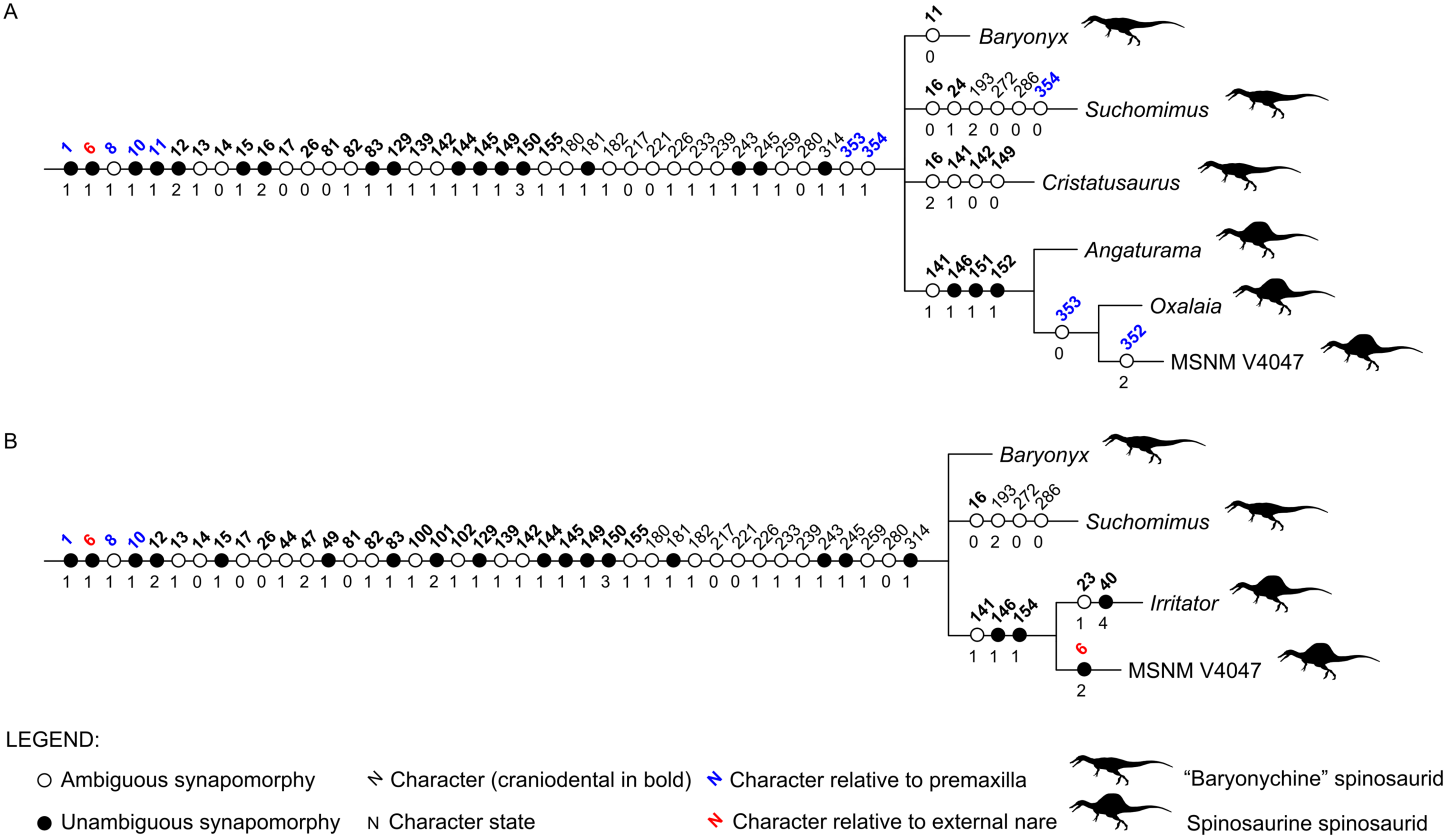
Synapomorphies of Spinosauridae recovered by the current analyses. A, Ingroup relationships after the cladistic analysis which excludes *Irritator*. B, Ingroup relationships after the cladistic analysis which excludes *Cristatusaurus*, *Angaturama* and *Oxalaia*. Silhouettes modified from Sales et al. [37,71].

Our cladistic results are shown in Figs 7–10. Firstly, the analysis of the whole matrix (S1 File) resulted in 145 most parsimonious trees (MPTs) whose strict consensus tree (1069 steps; consistency index, CI = 0.409; retention index, RI = 0.666) is shown in Fig 7. In general, the consensus topology is somehow comparable to that presented by Carrano et al. [29] and Evers et al. [17], except for the higher amount of polytomies (especially within the clade Megalosauria). The ingroup relationships of Spinosauridae were recovered as a polytomy formed by all spinosaurids OTUs, similar to Evers et al. [17].

Alternatively, the analysis focusing on spinosaurids preserving premaxillae, i.e., excluding *Irritator*, resulted in 150 MPTs. The strict consensus between them (Figs 8 and 10A; 1051 steps; CI = 0.415; RI = 0.670) shows a different topology within Spinosauridae. *Baryonyx*, *Cristatusaurus*, and *Suchomimus* form a polytomy along with the clade including the remaining spinosaurid OTUs. This seems to be mainly due to the variable placement of *Cristatusaurus* in different trees, as it sometimes appears as the sister group of either the remaining spinosaurids or the clade formed by spinosaurine spinosaurids. However, no single tree recovered *Cristatusaurus* and *Suchomimus* as sister groups of each other. Within the spinosaurine clade, the Brazilian taxa are successive outgroups of MSNM V4047.

Lastly, the analysis including spinosaurids preserving external nares—excluding *Angaturama*, *Cristatusaurus*, and *Oxalaia*—found 148 MPTs. The recovered strict consensus (Figs 9 and 10B; 1056 steps; CI = 0.413; RI = 0.664) also presents a polytomy constituted by *Baryonyx*, *Suchomimus*, and the clade formed by the spinosaurines *Irritator* and MSNM V4047.

## Discussion

### Taxonomy of Spinosauridae

Over the years the following taxa have been proposed or often regarded as spinosaurid theropods: *Angaturama limai*, *Baryonyx walkeri*, *Cristatusaurus lapparenti*, *Ichthyovenator laocensis*, *Irritator challengeri*, *Ostafrikasaurus crassiserratus*, *Oxalaia quilombensis*, *Siamosaurus suteethorni*, *Sigilmassasaurus brevicolis*, *Spinosaurus aegyptiacus*, *S*. *maroccanus*, and *Suchomimus tenerensis* [1,2–4,8,15–17,19,21,26–28,62]. *Ostafrikasaurus* was erected based on isolated teeth from the Late Jurassic of Tendaguru and these teeth are morphologically different from Cretaceous spinosaurid teeth [1,21]. Among the remaining taxa, some are considered *nomina dubia*. For example, *Siamosaurus* is another spinosaurid genus based on isolated teeth that cannot be confidently set apart from most Cretaceous spinosaurid teeth, including those from Asia attributed to *Sinopliosaurus fusuiensis* [1,14,27]. These teeth may turn out to be referable to *Ichthyovenator*-like spinosaurs [15]. The practice of naming theropod species solely based on isolated teeth has been historically problematic and most of them were later shown to be invalid [1,29].

Other cases of *nomina dubia* include the aforementioned African taxa *S*. *maroccanus* and *Cristatusaurus*. Despite the invalidity of *S*. *maroccanus* and its possible synonymy with *S*. *aegyptiacus*, material assigned to these taxa (i.e., specimens MNHN SAM 124 and MSNM V4047) are clearly not referable to any of the Brazilian taxa. For instance, *Angaturama* is unique in bearing a well-developed sagittal premaxillary crest and an anterodorsal protuberance in the anterior margins of its premaxillae. Once suggested to be congeneric with *Spinosaurus* [15], the external nares of *Irritator* are in a more anterior location than those of MSNM V4047. Also, its nares are delimited by premaxillae, maxillae, and nasals, whereas in MSNM V4047 these structures are only bounded by the latter two bones. In fact, each spinosaurid taxon preserving the external nares presents a particular relationship between the bones around the nares, which is likely a solid character for diagnostic purposes (Fig 4). The African materials can also be differentiated from *Oxalaia*, given that the latter has premaxillae more oval-shaped in dorsal view than the former, along with additional differences. Considering the similarities between MNHN SAM 124 and MSNM V4047, these specimens may belong to the same taxon.

The reassessment of the Brazilian material also bears some implications concerning the taxonomic status of *Cristatusarus*, a taxon that may be a *nomen dubium* and/or a synonym of *Suchomimus* [4,28,29,62]. For example, both Nigerien taxa possess a dorsal sagittal premaxillary rim. Nevertheless, *Cristatusaurus* presents a well-convex secondary palate, which appears below the premaxillary tooth row in lateral view [11,62]. This condition is also seen in *Oxalaia*, *Baryonyx*, and the African spinosaurines (Fig 4A and 4C), and possibly in *Irritator* and *Angaturama* [3,5,28,62]. On the other hand, the convex palate of *Suchomimus* can only be seen in lateral view through breaks in the referred rostrum MNN GDF501 (Fig 4B) [4]. In addition, the preserved portion of the ascending process of the maxilla seems to be more slender in *Cristatusaurus* than in *Suchomimus*, although this may be due to the juvenile condition of the holotype of the former taxon (MNHN GDF 366) [4,62]. Thus, future analyses on the validity of *Cristatusaurus* should also focus on the taxonomic significance of these differences in relation to *Suchomimus*.

#### *Irritator* versus *Angaturama*

Another controversial issue in spinosaurid taxonomy is the possible synonymy between *Irritator* and *Angaturama* [3–5,26,50]. Authors who argue in favor of this scenario highlight the facts that both taxa come from the same deposit (the Romualdo Formation of the Araripe Basin) and that their holotypes are non-overlapping and/or complementary to each other. In addition, the dorsal sagittal crest of *Irritator* might have extended anteriorly in a way similar to the crest of *Angaturama*. This could suggest that both specimens were part of the same skull [4,50]. In this case, *Irritator* would have priority over *Angaturama*.

However, supporting the idea of both holotypes being part of a single specimen has been a much more speculative practice than a substantiated argumentation. If they did not overlap in relation to any part of their preserved cranial remains, it would be impossible to demonstrate either the morphological similarities or differences between *Irritator* and *Angaturama*. In fact, the supposed non-overlap between these taxa would hamper any assertion on their possible synonymy.

**Fig 11:**
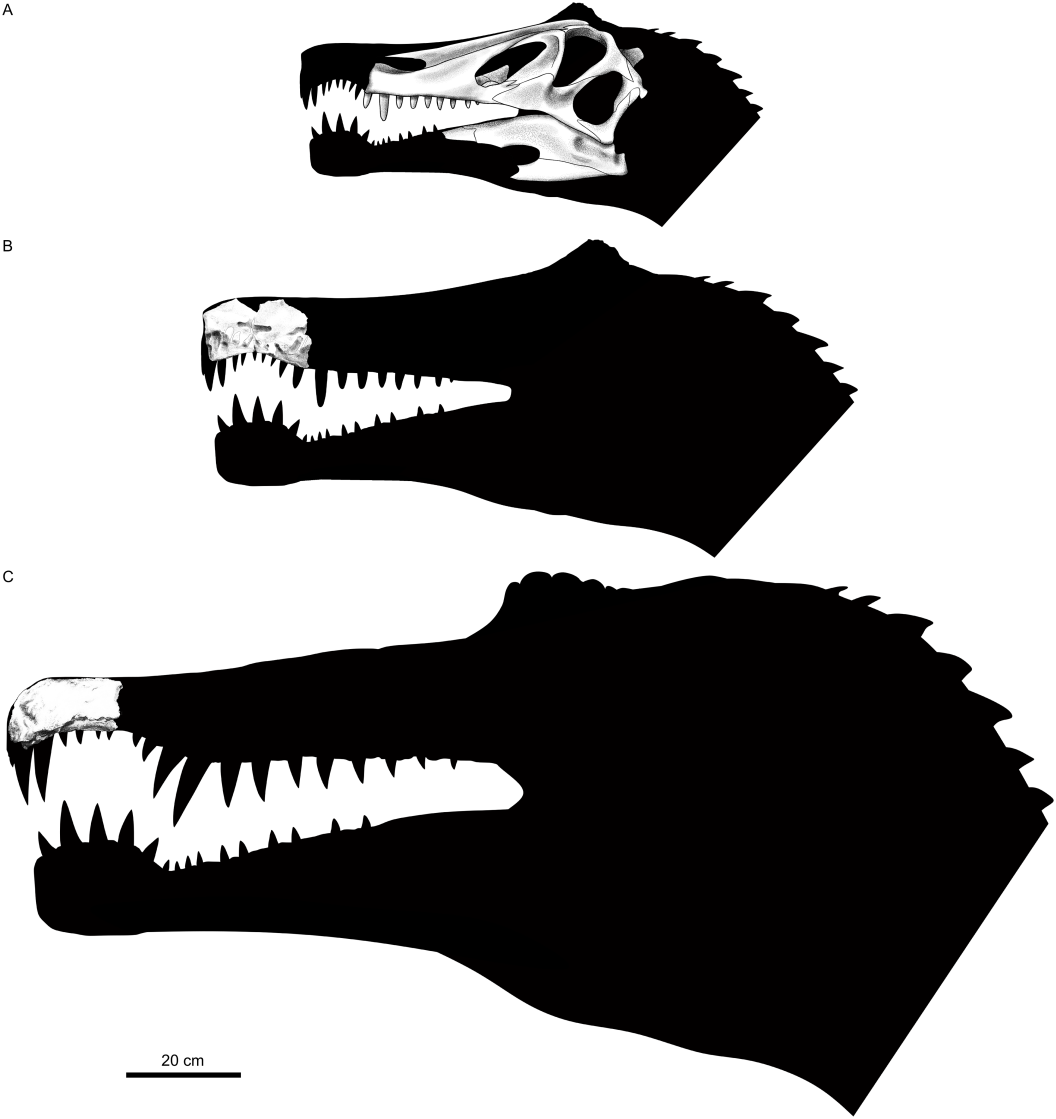
Spinosaurid cranial remains from Brazil. A, Reconstruction of specimen SMNS 58022, the holotype of *Irritator challengeri*. B, Specimen USP GP/2T-5, the holotype of *Angaturama limai*. C, Specimen MN 6117-V, the holotype of *Oxalaia quilombensis*. The fragmentary maxilla (MN 6119-V) tentatively referred to *O*. *quilombensis* was not included. A is modified from Sues et al. [15].

Nevertheless, the reinterpretation of certain features in both taxa sheds new light on this issue. Each holotype preserves tooth Lm3, which indicates that the specimens overlap minimally and cannot be considered at the very least the same individual. An opposite statement would require the reidentification of the assumed Lm3 as another tooth in any of those taxa. This is not possible in *Angaturama*, as it preserves the anteriormost portion of the tooth row, enabling the counting of the left maxillary teeth from the first functional alveolus to the last alveolus of a replacement tooth, posterior to the third functional alveolus (Fig 5). On the other hand, for a reinterpretation of tooth Lm3 of *Irritator*, it is necessary to demonstrate that the size variation pattern of its maxillary teeth does not follow that seen in *Baryonyx*, *Suchomimus*, MNHN SAM 124, and MSNM V4047. However, the pattern seen in maxillary teeth of *Irritator* matches that found in the aforementioned taxa and specimens (Fig 3). Thus, the evidence at hand supports that the holotypes of *Angaturama* and *Irritator* each preserve tooth Lm3 (Fig 11).

A complementary approach is to consider the preservation conditions of each specimen. The right side is considered the best preserved in both holotypes [15,19,49], but some differences can be highlighted. Firstly, they differ in color, as the holotype of *Irritator* is brighter than *Angaturama* (Figs 2 and 5). They also differ regarding the amount and type of damage. The left side of *Angaturama* is more affected by cavities filled by recrystallized calcite (Fig 5A), whereas the left side of *Irritator* shows this mineral filling in a large vertical break just behind the external nares (Figs 2A and 3A). The most obvious difference involves the preservation of the dentition. *Angaturama* lacks some teeth and those preserved in situ are usually damaged, being longitudinally or transversely cross-sectioned, which seems to be related to the loss of the ventral borders of the tooth row (Fig 5A and 5B). Only tooth Rpm6 has the enamel of both sides preserved, showing that only the lingual one was fluted [19,26]. However, the teeth of *Irritator* are, in general, well preserved in situ; few of them are as damaged as those of the other taxon (Figs 2 and 3). Most teeth of *Irritator* are labially fluted and some of them present marginal transversal undulations (Fig 3) [15]. Furthermore, Machado and Kellner [34] stated that the posterior part of the holotype of *Angaturama* was more laterally compressed than that of *Irritator*, indicating that they were not part of the same concretion. This is also supported by Kellner et al. [28], but they did not justify their claim.

Despite being significant, these differences do not imply that the specimens belong to different individuals. Some fossils from the Romualdo Formation were affected by various taphonomic processes throughout their concretions, especially fractures, calcite recrystallization, and different degrees of compression. Also, having likely undergone different preparation techniques, the extension of damages would be unique for each specimen, which could explain the different colors and degrees of preservation of the dentition, along with other attributes. Even the difference in tooth ornamentation is not informative in this regard, given that this feature varies within the tooth row of a single taxon [72]. Only the preservation of tooth Lm3 by both holotypes precludes their attribution to the same individual, although it does not clarify if they are synonyms. Furthermore, the holotypic premaxillae of *Angaturama* and *Baryonyx* are comparable in size, which indicates that the complete skull of the holotype of *Angaturama* might have been larger than that of *Irritator* (Fig 11) [3,15,19,70].

In short, the holotypes of *Irritator* and *Angaturama* likely belonged to different individuals, but the hypothesis of considering them as synonyms requires additional overlapping cranial remains and further study for either its refutation or corroboration.

### Evolution of craniodental features

Spinosauridae are frequently split into two subclades, Baryonychinae and Spinosaurinae (e.g., [1,4,5,16,29,64,73]). This dichotomy is supported mainly by craniodental characters, which involve the biggest amount of overlapping features between genera within each subfamily. For example, Baryonychinae are usually characterized by finely serrated and more mesio-distally curved tooth crowns that are separated by less marked diastemata in premaxillae. On the other hand, Spinosaurinae have unserrated and straighter, conical tooth crowns that are partially separated by clear diastemata in premaxillae. In addition, the number of maxillary teeth of baryonychine spinosaurs is almost twice as much as that of spinosaurines. The third main craniodental feature distinguishing these subclades involves the position of the external nares: they are more anteriorly positioned in baryonychines than in spinosaurines [1,4,5].

Recently, Evers et al. [17] presented a polytomy formed by spinosaurid OTUs, similar to the results of our first cladistic analysis (Fig 7). Although these results may be due to a large amount of missing data (especially regarding postcranial characters), it leaves open the possibility of a nondichotomous phylogenetic relationship between baryonychine and spinosaurine spinosaurids. This appears likely in the consensus of our second and third analyses (Figs 8–10), in which typically baryonychine taxa do not form a monophyletic clade. This hypothesis is supported by the fact that many baryonychine features correspond to intermediate stages between the plesiomorphic theropod and the derived spinosaurine states of some characters. This can be illustrated by the probable evolutionary history of the serrations in spinosaurid dentition: theropods initially had coarsely serrated teeth, which might have then become finely serrated, as in baryonychines, and finally unserrated in spinosaurines [21–23]. Such a scenario suggests that, in the future, baryonychines may be shown to correspond to successive outgroups of Spinosaurinae, although the latter subfamily may be indeed monophyletic. In this sense, Baryonychinae would be paraphyletic, as is typically the case for groups characterized by plesiomorphies (e.g., non-avian dinosaurs itself and “rhamphorhynchoid” pterosaurs [68,74,75]).

The provided cladistic framework and reinterpretation of certain spinosaurid features are compatible with the aforementioned evolutionary scenario, at least with regards to some craniodental features (Fig 10). For example, the plesiomorphic condition of theropod premaxillae is the presence of five or less teeth. The increasing tooth number within Spinosauridae and their arrangement in a terminal rosette might have occurred prior to the appearance of well-marked diastemata in the premaxillary tooth row of Spinosaurinae. The plesiomorphic condition of the spinosaurid premaxillae is possibly the possession of a dorsal sagittal crest, as it is present in both baryonychines and in the spinosaurine *Angaturama*. This feature later disappeared at some point within Spinosaurinae. These evolutionary scenarios are supported by our second strict consensus tree, in which *Angaturama* is the sister-group of *Oxalaia* and MSNM V4047 (Figs 8 and 10A). In all aspects, *Oxalaia* is much more similar to the African spinosaurines than *Angaturama* (Figs 4 and 11).

Considering the particular pattern of tooth size variation shared by baryonychines and spinosaurines, the most parsimonious hypothesis is that the maxillary dentition is homologous within Spinosauridae, with regards to the first (mesial-most) four teeth and corresponding alveoli. However, the higher number of maxillary teeth in baryonychines than in spinosaurines implies that some teeth were lost during the evolutionary history of Spinosauridae. This casts doubt on the homology between dental elements behind m4 of those groups, where the loss of teeth likely took place. Accordingly, Dal Sasso et al. [5] stated that, in spinosaurine spinosaurids, maxillary teeth are usually separated from each other by a space equivalent to the size of one maxillary alveolus and that this pattern starts just behind m4, suggesting an intercalated loss of teeth. For instance, based on the 22 maxillary teeth of *Suchomimus*, the hypotheses above would predict a number of 13 maxillary teeth in spinosaurines, if the intercalated loss took place just behind m4. The spinosaurine MSNM V4047 had at least 12 maxillary alveoli, whereas the holotype of *Irritator* is inferred to have 11. This difference between the hypothetical and the observed numbers of maxillary teeth may be explained by the closure of some alveoli during ontogeny or due to the above-mentioned reasons. Another fact is that the last maxillary teeth of both MSNM V4047 and *Irritator* are placed at the level of the anterior borders of the antorbital fenestrae, whereas the maxillary tooth row of *Suchomimus* extends much more posteriorly (Fig 4). Thus, it is probable that, along with the intercalated loss of maxillary teeth likely behind m4, the reduction of maxillary teeth in spinosaurine spinosaurids was caused by the loss of the last alveoli placed posteriorly to the beginning of the antorbital fenestrae.

The evolution of the spinosaurid external nares also deserves consideration. They are clearly placed in a more posterior location than in any other theropod taxon. Contrary to previous assumptions [1,4,5], within Spinosauridae, a more anterior location of the nares is shared by baryonychines and the spinosaurine *Irritator* (Figs 4 and 11B). This condition might have allowed the participation of premaxillae, maxillae, and nasals in surrounding the external nares of those spinosaurids. During the transition of the external nares to a more posterior location, the participation of premaxillae in their anterior and ventral portions likely became progressively less important until reaching the condition seen in MSNM V4047, whose external nares are only surrounded by maxillae and nasals. Also, the external nares seem to become progressively smaller within Spinosauridae. In relation to the antorbital fossae and fenestrae, *Irritator* also presents the intermediate relative and absolute sizes of the external nares, whereas the largest and the smallest ones are found in baryonychines and MSNM V4047, respectively (Fig 4). This feature does not seem to be related to skull length, as *Irritator* has the smallest skull.

In general, the spinosaurid cranial remains from the Araripe Basin are somehow intermediate between the morphology of the skulls of baryonychines and the remaining spinosaurines, which resonates with their phylogenetic relationships (Figs 8–10). Kellner et al. [28] also considered Araripe spinosaurids as being very different from other spinosaurine cranial remains. Furthermore, *Oxalaia* is clearly more related to the African spinosaurines (Figs 8 and 10A). Hence, at least with respect to *Angaturama* and *Oxalaia*, the Brazilian spinosaurid taxa represent successive outgroups to the African spinosaurine material MSNM V4047 (and MNHN SAM 124). Spinosaurinae seem to have been more morphologically diverse than previously thought.

#### Palaeobiological significance

Multiple craniodental features of Spinosauridae were likely related to semiaquatic habits. These include: conical teeth, laterally compressed rostra, and retracted external nares [1,3,7,11,15,64,76,77]. The isotopic composition and histological pattern of various spinosaurid teeth and bones, respectively, also resembled those of semiaquatic to aquatic taxa [6,7]. These habits must have allowed these dinosaurs to become more associated with coastal environments than other coeval theropod groups, such as Abelisauridae and Carcharodontosauridae [71].

However, the isotopic data also suggest that some spinosaurids might have been more terrestrial than others [1,6]; this could have been an important niche partitioning ecological mechanism in the case of sympatric taxa. Varying degrees of association with water environments are also indicated by the histological sections of limb bones of *Suchomimus* and the proposed neotype of *S*. *aegyptiacus*. The former seems to have been a less aquatic animal than the latter [7].

This diversity of ecological roles among spinosaurid theropods might also have been related to the differing conditions of the external nares. As aforementioned, within Spinosauridae, these structures vary in both size and position. Proportionally smaller external nares suggest reduction of the relative importance of the olfaction for general behavior, whereas the larger ones suggest the opposite. Among spinosaurids, the smallest nares are those of MSNM V4047, which are also the most posteriorly placed. A more posterior location of the external nares (and, consequently, the fleshy nostrils) does not facilitate the collection of olfactory cues [78]. Another approach is to consider the amount of space between the external nares and the olfactory bulbs, which comprises the nasal cavity and is indirect evidence of the available surface for the olfactory epithelium [79]. In birds and crocodilians (the extant phylogenetic brackets of all non-avian dinosaurs), the bulbs are delimited laterally by the crista cranii [80]. In *Irritator*, the crista cranii are placed between the orbital cavities (Fig 2) [15] so that the olfactory bulbs must have terminated at the level of the anterior half of the orbits, which might have been the same for other spinosaurids. Based on the proposed reconstructions of the skulls of *Suchomimus*, *Irritator*, and MSNM V4047, the latter seems to be the spinosaurid with the proportionally smallest space for the nasal cavities and, consequently, the olfactory epithelium [4,5,7,15]. One could argue that a smaller space could be compensated by a greater concentration of olfactory sensory cells, but such a hypothesis is untestable [79].

Olfaction is not the main sensory source of (partially or mostly) piscivorous predators, especially some amniotes. In these cases, visual and nonvisual sensory systems other than olfaction are of major importance [81–85]. For example, crocodilians have mechanoreceptors on their snouts which sense the movement of the water surface and are useful in low-light conditions, whereas birds are mainly visual predators [84,85]. Vision was also important for pterosaurs and might have played a significant role during fishing [86]. The premaxillae of spinosaurids display many foramina which were interconnected and formed extensive internal channels. These structures are reminiscent of the crocodilian system of mechanoreception and might have had an analogous sensory function [7]. Thus, MSNM V4047 might have relied more on mechanoreception than on olfaction, whereas the latter sense might have been more important for the predatory habits of *Irritator* and baryonychines than for those of the African spinosaurine.

The fossil record, here assumed to be mainly episodic and to have preserved, in general, the most frequent paleobiotic condition, somehow supports the hypothesis above. The holotype of *Baryonyx* includes partially digested fish scales and a limb bone of a juvenile ornithopod in its rib cage [3]. Also, a mechanical profile proposed for the lower jaw of *Suchomimus* suggests that this taxon could also have preyed on small terrestrial animals [87]. In addition, a series of three cervical vertebrae of a pterosaur from the Romualdo Formation of the Araripe Basin has a spinosaurine tooth crown embedded in it, indicating that some spinosaur from that region also fed on non-aquatic prey [33,88]. On the other hand, a partial vertebra tentatively referred to the sclerorhynchiform *Onchopristis* was found associated with a tooth alveolus of MSNM V4047 [5]. Although this finding might be regarded at best as a dubious evidence of piscivory, the morphology of that spinosaurid snout suggests that fish was a food resource for spinosaurids such as MSNM V4047. Moreover, the current reconstruction of the skeleton of *S*. *aegyptiacus* based on the proposed neotype also supports that semiaquatic habits might have been well developed in some spinosaurids, possibly including the feeding behavior [5,7,64]. The evidence at hand indicates that baryonychines and some spinosaurine from the Araripe Basin also included terrestrial prey in their diet, for which olfaction might have been more useful. However, MSNM V4047 may have mainly fed on aquatic items such as fish, relying possibly on vision and mechanoreception for capturing its prey.

The spinosaurid dentition is indicative of a partially piscivorous diet. The conical tooth crowns mirror those of crocodilians and other piscivores and were ideal for seizing most fish [3,89]. Also, the upper and lower terminal rosettes must have been the most appropriate portions of the spinosaurid jaws for grasping aquatic prey [87,90]. Accordingly, the tip of the upper jaw is well perforated by the likely sensory foramina, indicating that at least its anterior portion might have been submerged while the animal foraged in water [5,7]. In this sense, the retracted nares enabled breathing at the water-air interface as they were likely kept above the water surface during foraging [1,7]. This might be additional evidence that olfaction did not play a significant role in aquatic prey detection [90]. However, the exact functional significance of the reduction in number of maxillary teeth within Spinosauridae is less clear. The pattern regarding the size variation of the first maxillary teeth suggests that some factor or pressure was responsible for its maintenance in different taxa. On the other hand, the reduction of maxillary teeth, including the possible intercalated loss of some of them, happened independently from the retraction of the external nares, as *Irritator* presents almost as few teeth as MSNM V4047 but has more anteriorly placed nares than the latter (Fig 4C and 4D). New fossil remains and interdisciplinary studies are mandatory for elucidating this issue and others, including additional biomechanical approaches [90].

## Final remarks

Spinosaurid dinosaurs have been surrounded by an enigmatic atmosphere due to their bizarre anatomy and morphology as well as the fragmentary nature of most collected specimens [1]. The tragic loss of the holotype of *Spinosaurus aegyptiacus* also contributed to this condition [5,9,91]. Despite being incomplete, spinosaurid cranial remains from Brazil are informative in multiple ways and help fill in some gaps in the knowledge on these theropods. The reinterpretation of certain craniodental features supports, for example, the distinction between *Irritator* and *Angaturama* at least at the individual level, and our cladistic results indicate the latter taxon and *Oxalaia* as successive outgroups of MSNM V4047. In addition, the evolution of spinosaurid craniodental features is likely related to different trends towards semiaquatic and/or piscivorous habits. These trends might have had a major impact on the position and size of the external nares, suggesting the reduction of the importance of olfaction in relation to other senses during foraging. However, other issues remain more disputable, such as the possible non-monophyly of Baryonychinae, the possible synonymy between the Araripe spinosaurids, and the sequence of morphological changes during the evolutionary history of Spinosauridae. Further study is needed to address these issues, including the formal description of additional cranial and postcranial remains (e.g., [41]). In this sense, although African materials are usually the focus of most investigations, Brazilian specimens play an important role in discussions concerning the evolution and paleobiology of Spinosauridae. This statement is clearly corroborated by new findings of these dinosaurs in understudied fossil sites (e.g., [37,54]).

## Supporting information

S1 File Supporting information on the character statement list and taxon-by-character matrix of the current study. (PDF)
