# Supplementary material for: Spinosaur taxonomy and evolution of craniodental features: Evidence from Brazil
Source: PLoS One. 2017 Nov 6;12(11):e0187070. doi: 10.1371/journal.pone.0187070 (PMC5673194; doi:10.1371/journal.pone.0187070)
Supplement: S1 File — (PDF) [file pone.0187070.s001.pdf]

Supporting File of:

Spinosaur taxonomy and evolution of craniodental features: Evidence from Brazil

Marcos A. F. Sales[1*] and Cesar L. Schultz[1]

[1] Departamento de Paleontologia e Estratigrafia, Instituto de Geociências, Universidade Federal do Rio Grande do Sul (UFRGS), Porto Alegre, Rio Grande do Sul, Brazil

*Corresponding author. E-mail: marcos.paleo@yahoo.com.br

Modifications in the character statement list of Carrano et al. [1]

3. ***External naris****, delimiting bones: only pre-maxilla and nasal (0); pre-maxilla, maxilla, and nasal (1); only maxilla and nasal (2).*

Remarks: character statement modified.

6. ***External naris****, position of the anterior border in relation to the tooth row: at the pre-maxillary tooth row or at the edge (0); at the anterior half of the maxillary tooth row (1); at the posterior half of the maxillary tooth row (2).*

Remarks: character statement modified.

16. ***Maxilla****, position of palatal process: intermediary, immediately dorsal or almost parallel to paradental plates (0); dorsal, immediately ventral to dorsal surface of maxillary anterior ramus (1); ventral, just below the tooth row in lateral view (2).*

Remarks: character statement modified.

40. ***Nasal****, development of dorsolateral surfaces: none, nasals low and dorsally convex (0); pronounced dorsolateral rims, sometimes with lateral crests (1); tall, parasagittal crests (2); inflated and forming a hollow midline crest (3); mid-sagittal rim similar to a crest at or close to the posterior portion of the conjoined nasals (4).*

Remarks: new character state added (character state 4).

139. ***Paradental plates****, visibility in medial view: widely exposed, subpentagonal and moderate–tall (0); obscured by medial wall of the bone, triangular apices only may be visible (1).*

Remarks: character statement modified after Carrano and Sampson [2].

352. ***Pre-maxilla****, condition of the mediolateral constriction of the posterior portion, regarding the tooth row: width decreases progressively from pm3 to the last alveolus (0); width decreases progressively from pm3 to pm 5 and, then, steeply from pm 5 to pm6, at which it stabilizes in its narrowest dimension (1); width decreases steeply from pm3 to pm4, progressively from pm 4 to pm 5 and, then, steeply again from pm 5 to pm6, at which it stabilizes in its narrowest dimension (2); width decreases only after the entire tooth row (3).*

Remarks: new character statement added.

353. ***Pre-maxilla****, aspect of the dorsal surface: mostly convex but lacking a dorsal rim or crest (0); with a sagittal crest (1).*

Remarks: new character statement added.

354. ***Premaxilla****, ventral extent of the medial wall (and palatal portion) in relation to the tooth row or alveolar margin in lateral view: dorsal or at the same level, not visible in lateral view (0); ventral, visible in lateral view (1).*

Remarks: new character statement added.

Taxon-by-character matrix for the operational taxonomic units used in this study

Eoraptor 00010 000?0 0000? ????? 01010 00?0? 00100 00?00 00100 ??000 1?000 00010 00000 ?0000 00000 0?10? 00??0 ?0010 00?00 00?0? ????? 0??00 ?0?10 10000 00?0? 0000? 00?00 00?0? 00?00 01000 00000 00090 ????? ????8 00000 00000 0000? 0?000 00000 00000 0?000 0001? 00100 00?00 00010 00000 10100 00000 00?00 10000 01000 00000 00001 00000 00?00 00000 0000? ?000? 00000 00?00 000?0 000?0 00?00 00000 00??? 80000 00000 00000 00000 00000 0?0?

Herrerasaurus 00001 00000 0000? ???0? 00010 00?0? 0?000 00?00 00100 ??001 20100 00000 00000 ?0000 01000 00000 020?0 00010 ?0000 0?10? ?0000 0000? 00000 0?100 00?0? 0000? 00001 00100 00?0? 01000 00000 00080 0?000 010?9 00000 00000 0000? 0?100 0000? 00000 00000 000?? 001?? 00?1? 011?0 ??000 10100 00001 00000 10100 01000 00000 00001 00000 00?00 00000 0000? 0?001 00000 0?000 00??0 00010 00000 00000 001?? 90100 00100 00000 00000 00000 0?00

Acrocanthosaurus 020?1 000?0 00000 11110 00000 21111 11000 10?00 00002 11101 21110 00202 12011 ?1121 11102 0110? 02??1 10121 00000 10011 01000 12011 111?1 11002 10010 10200 10?11 111?0 00100 01000 00100 11012 ?0011 0011? 00?00 12110 00100 01100 11211 110?0 02100 00001 01?1? 11011 01200 12001 210?0 01011 11000 01131 10111 11100 0???? 010?1 0??11 10110 20120 1?120 ?2011 11000 22011 10111 10000 11001 0312? 1010? 1110? 1??01 21011 1001? 1?00

Aerosteon ????? ????? ????? ????? ????? ????? ????? ????? ????? ????? ????? ?01?0 00?11 1???? ????? ?1000 0200? ????? ????? ????? ????? ????? ????? ????? ????? ????? ????? ????? ????? ????? ????? 11012 1???? ???11 ?0100

1?1?0 00100 01011 01011 1???? ?2?11 0??0? ????? ???01 01100 121?? ?????
????? ????? ????? ????? ????? 01011 01001 02211 10110 20120 1?120 ?????
????? ????? ????? ????? ?1001 ?412? ???01 11202 1110? ????? ????? ????

Afrovenator ????? ????0 01010 11000 00100 12001 1000? ????0 ?0002 01001
?0110 01011 000?? ????? ??00? 00111 1001? ????? ????? ????? ????? ?????
????? ????? ????? ????? ????? ??000 00000 01??? ??10? 11001 ?0?01 10000
10001 10110 0001? 001?0 00001 ????? ??100 000?? ?1?01 010?? ????? ???01
21??? ??00? 0???? ?0?31 00111 1?100 001?1 0?011 01011 ?011? 2012? 1?00?
00101 11000 11101 10000 11000 01000 021?1 10101 10??? 11101 1?011 100??
1???

Allosaurus 00101 00000 01000 10100 00000 21111 11000 11011 00002
[01]2[01]00 10110 00000 01011 11100 01102 00000 02001 10110 00110 10010
01020 12011 11111 11200 01001 10201 11111 11100 00100 01102 00100 11001
10011 0010[01] 10000 10110 00100 00100 01011 11000 011?0 00000 0101? 11011
01200 12001 21010 11000 01000 11131 00111 11100 00111 01001 02111 10110
20120 1?110 02001 11000 [12]1011 10111 10000 11001 02122 10101 11102 11101
21011 10010 1?00

Angaturama 11??? ?0101 121?1 ????0 ????? ????? ???0? ????? ????? ?????
????? ????? ????? ????? ????? ????? ????? ????? ????? ????? ????? ?????
????? ????1 ?0??? ????? ????? ????? 11011 1?013 11??? ????? ????? ?????
????? ????? ????? ????? ????? ????? ????? ????? ????? ????? ????? ?????
????? ????? ????? ????? ????? ????? ????? ????? ????? ????? ????? ?????
????? ????? ????? ????? ????? ????? ????? ????? ????? ????? ????? ?????
??1?

Australovenator ????? ????? ????? ????? ????? ????? ????? ????? ????? ?????
????? ????? ????? ????? ????? ????? ????? ????? ????? ????? ????? ?????
????? ????? ?0000 1???? ????? ??100 00100 0110? ????? ????? ????? ?????
????? ????? ????? ????? ????? ????? ????? ????0 ????? ????? ????? ?????
????? ???00 01010 1???? 01?11 11??1 01??? ????? ????? ????? ????? ?????

????? ????? 22012 10111 10001 11001 14122 10101 11212 11101 211?? ??010 ????

Baryonyx 111?0 10101 02101 20?00 00??? 02??? 1??01 ????0 ?0012 02011 ?0??? ???0? ?0?11 11100 ????? ?0?11 01101 00010 11?01 ??010 210?0 11??? ????? ??001 10101 0?[12]10 1???1 ??010 01011 00013 00101 1100[12] ?1001 00100 10001 10111 11010 00110 00101 010?0 ???00 0?00? ?1??1 ?00?? 0?1?0 10001 21111 00010 01101 0???? ????? ???10 ?0?11 010?1 01?11 10110 [01]0?2? 1?0?? ??001 1000? ???1? ???00 11010 0???? ????1 10?0? ????? ????? ??0?? ????? ?011

Carcharodontosaurus ????? ?0??0 00000 11110 00010 01101 11010 11110 00?02 ?1?0? ?1111 00??2 12112 11?21 1???? 0???? ????1 10121 ?0??0 100?? 01121 121?? ?1??? ????2 ?10?? ????? ????? ??100 00200 00??? ??10? 110?? ????? 0?10? 0?0?1 ???0? ????? ????? ????? ????? ???10 0?0?? ?1??? 1???? ????? ????? ????? ????? ????? ????? ????? ????? ????? ?1??1 ????? ??11? ????0 ????? 0??11 ????? 22??1 10111 1??01 ?100? ????? ?010? ????? ????? ????? ????? ????

Ceratosaurus 02101 00010 00000 00000 00000 01010 00101 20?00 00002 02001 10000 00010 00011 11110 00101 00100 10001 01010 00000 00110 10000 11100 00?10 00100 00001 ??100 00001 00100 01000 00101 00100 01102 1?010 00100 00000 02110 00000 10100 00101 0211? ?1000 0210? 01000 01101 01111 01001 00110 000?? ??0?? 1??1? 01?00 010?0 10?11 100?0 00011 10111 10110 10000 1[01]000 10011 00110 [01]0011 02100 01010 02002 11101 00102 1??1? 0001? ?10?? ??00

Chilantaisaurus ????? ???01 20110 000?? ????? ????? ????? ???11 ????? ?10?? ??211 0???? ????? ?????

????? ????? 22??1 11??? 1000? 1100? ?4122 1???? 1???? ????? 2101? ?0??? ????

Chuandongocoelurus ????? ???0? ????? ????? ????? ????0 ????? ????? ???0? ????? ???0? ????? ????? ????? ????? ????? ????? ????? ????? ????? ????? ????? 00??? 00111 01011 ???1? 0???? ?0??? ????? ????? 00?10 10001 00000 ?1000 041?2 12101 1???2 1110? 100?? 100?? ????

Coelophysis_bauri 00101 01100 0010? ???0? 01101 0100? 00000 00?00 00101 ??000 00000 00000 000?0 ?0000 01101 10100 000?0 00000 0?0?2 ??1?? ??0?? 1?0?0 ?0?00 ??001 10?0? 0000? 00?00 00000 01000 0?000 00000 01100 ?1000 11000 ?0000 01011 00000 0?000 00000 01000 00000 0011? 1100? 01?00 00010 02000 10100 0000? 0[01]?00 10111 01010 00000 10010 00110 00011 01111 0100? 10000 00100 00100 00110 [01]00?0 0?000 ?0100 01100 02100 00001 01111 00011 00000 0300

Coelophysis_rhodesiensis 00??? 01100 0010? ??00? 01101 100?0 00000 00?02 00101 00001 [01]0000 0000? 00010 00000 00?01 10?00 00000 00000 00002 00100 ?000? 10000 00000 10001 00000 00000 00000 00100 00000 01000 00000 01101 1?000 11000 00000 01011 00000 00?00 00000 01000 0?000 0?11? 11000 01?00 00010 02000 10100 0?001 0[01]000 11011 01010 00000 10010 00110 00111 01111 0000? 1000? 01110 00100 00111 [01]0011 02000 ?0100 ?1100 02100 00001 01111 00011 01000 0300

Compsognathus 00??? 00000 0100? ???0? 01000 2100? 1??00 00?00 00000 ??000 00?00 0?00? 00??? ?1?00 ????? ???0? ?0??? ?0??0 0???? ?0??0 ?0??? ???1? 1???? 1?000 0?00? ?0??1 00??1 0100? 0000? 0??00 00100 ?100[12] ????? ??100 ?001? ?0011 00?00 0???? 0000? ?1??0 ??000 ?0?00 ?1?0? 0111? 01110 0?0?? [12]??0? ??0?? ?0??0 1?131 0011? ???0? 000?? 010?1 1??11 0011? 20?20 1?011

?11?1 0??00 2???1 11??? ????? ?1?0? ????? ?0101 1???? ???01 ??0?1 1?010 1?00

Concavenator ????? 0???? ???0? ????? 00100 2??0? 1?00? 1111? 10102 01000 0?110 ??10? ?2??? ?1??? ????? ????? ????? ????? ????? ????? ????? ????? ????? ????? ????? ????? ????? ????? 1020? 0???? ??[01]0? ?100? ????? ????? ????? ?[12]?1? ?10?? ??1?? ?0211 ?1?1? ?[01]100 ?000? ???1? 11111 0?10? 1??0? 1??10 ??0?1 01000 1???? ????? ????? 0?0?0 ?10?1 1??11 10110 20??? ????? 02011 ???1? 22??1 100?? ????? ????? ??[12]2? ?0?0? 1?1?? ???0? ??0?1 ?00?? ??0?

Condorraptor ????? 00100 01??? ????? 01001 ????? ???00 00?00 101?0 00010 00?20 01?01 11010 01?00 02??? 01??1 01??? ????? ????? ????? ????? ????? ????? ????? ????? 0?1?? 01?01 010?? ????? ?0??? ?0??? 00001 100?? ????? ?0??0 10000 01?00 ??11? ????? ????? ????? 1?01? ??0?? ????

Cristatusaurus 11??? ??1?1 1??01 2??00 0???? ?20?? ???0? ????? ????? ????? ????? ????? ????? ????? ????? ????? ????? ????? ????? ????? ????? ????? ????? ????? ???0? ????? ????? ??010 10?11 ??003 00??? ????? ?011

Cryolophosaurus ????? ????? ????? ????0 0???? ????? 1??00 ?1001 ?0101 0?000 ?0111 ??0?0 0[01]0?1 ?1000 0?10? ?0000 00??1 00010 00001 ??1?0 010?? ??0?? ????1 ??0?? ????? ????? 10?00 00000 00?0? 01??? ???0? 01000 ????? ???00 ?0?00 0?0?1 000?? 00100 0??01 0?0?? ???00 0200? ???0? 01??? ????? ????? ????? ????0 ?1000 1???? ????? ????? 00??? 00??? 0???? ?011? ?0??? ?0???

0?0?? 10011 001?0 ?0011 01000 00000 ?2??? ???01 00101 1??0? ????? ????? ????

“Dilophosaurus” sinensis ?0?0? 000?0 010?? ????? 00020 2??0? 1??0? ?1013 00100 ??000 ?00?0 0?01? 000?? ?1??0 ????? 1?0?? ????? ????? ????? ????? ????? ????? ????? ????0 0??0? 00??? 1???? ????? 0??0? 0???? ??10? 0100[12] ?1?00 ??000 10000 ?0010 00000 100?0 00001 ??000 ?1000 020?? 01?01 0??01 0001? 00011 21110 00000 01000 1???1 01011 1?000 000?0 101?0 00011 1011? [01]0020 10000 ?2000 10111 00111 00001 01000 ?1000 02200 1010? 0???? 0???1 10011 100?? 1?00

Dilophosaurus 01?11 01100 00100 0000[01] 00100 01000 00000 ?1002 00100 ??00? 20010 0001? 00010 01000 ?1101 10100 00000 00?00 00002 00101 00000 1??0? ?0??? ??201 10001 0000? 10000 00[01]10 00000 01000 00101 01101 ??000 11100 10000 01011 00000 00000 00101 01000 01000 0201? ?1000 01000 00010 01001 10100 00001 01000 1?111 01100 01000 10010 00110 00011 00110 [01]000? 10000 01000 1?001 00110 [01]0000 00000 00100 02110 02100 00101 01101 00011 000?0 ?301

Dubreuillosaurus 01??? 000?0 01010 1?000 00100 1[12]000 1??0? ?0??0 ??002 ??001 ?0??? ?1011 ?0010 11?00 0000? ????? ????1 0??20 11100 ?0??1 10010 ??0?? ????0 10?01 10111 ??201 ????? ??000 0000? 01000 00100 ?1001 ????? ????0 ????? ????0 ?00?? 0?1?? ????1 ????? ??1?? 000?1 0???? 0110? ????0 ????? ????? ????? ????? ????? ????? ????? ????? ????? ????? ????? ????? ????? ????? ????? ????1 1???? 0?0?? ????? ????1 ?01?? ????? ????? ????1 ????? ??00

Duriavenator 00??? 000?? 01010 11000 ?0?0? 120?1 ???0? ????? ????? ????? ????? ????? ????? ????? ????? ????? ????? ????? ????? ????? ????? ????? ????? ????1 10101 ????? ????? ??000 0010? 0??0? 00100 ?????

????? ????? ????? ????? ????? ????? ????? ????? ????? ????? ????? ?????
???0

Elaphrosaurus ????? ????? ????? ????? ????? ????? ????? ????? ????? ?????
????? ????? ????? ????? ????? ????? ????? ????? ????? ????? ????? ?????
????? ????? ????? ????? ????? ????? ????? ????? ????? 0110[12] ????? ???00
10000 0?011 00000 1?000 00000 02111 ?0000 011?? 010?? ????? 0???1 1?011
10100 00100 0???? ????? ????? ????? 00011 10000 00?11 11111 00?[12]? ?????
0?0?0 00011 00110 ?0011 0??00 01000 0210? ?21?0 ????? ?111? 001?? ?1???
????

Eocarcharia ????? ????0 00000 11100 10100 21111 11?0? ????? ????? ?????
????? ?01?0 02011 11??? 1???? ????? ????? ????? ????? ????? ????? ?????
????? ????? ????? ????? ????? ??100 0010? 01??? ??10? ????? ????? ?????
????? ????? ????? ????? ????? ????? ????? ????? ????? ????? ????? ?????
????? ????? ????? ????? ????? ????? ????? ????? ????? ????? ????? ?????
????? ????? ????? ????? ????? ????? ????? ????? ????? ????? ????? ?????
????

Eustreptospondylus ?1??? 00000 011?0 11000 00010 120?1 ???0? ????? ?1002
00001 ????? ?10?1 0001? 1??00 000?? ?0111 10001 0??10 01000 100?1 10000
12??? ????? ??001 10111 ????? ????? ??000 00?0? 01000 00100 11001 ???01
10100 10001 10110 00001 00110 00101 ?100? ?1?00 00??? ?1??? ????? 111?0
???01 21210 ?00?? ????? ????? ????? ????? ?0011 01011 00011 ?0113 00?2?
1?000 00101 0100? 11111 10000 11000 01000 02111 101?1 10102 111?? 1001?
?000? ??00

Fukuiraptor ????? ????? ????? ????0 ?0?0? ????? ????? ????? ????? ?????
????? ????? ????? ????? ????? ????? ????? ????? ????? ????? ????? ?????
????? ????? 0???? ????? ????? ??100 0010? 0110? ???00 110?[12] ????? ???1?
????? ????0 ????0 ????? ???11 ????? ????? ????? ????? ????? ????? 12001
21110 1?001 ?1010 ????? ????? ????1 0???? ?10?? ????? ????? ????? ?????

????? ????? 21012 101?1 10001 1??01 ??12? ???01 11212 1??01 2?1?? ?0??? ????

Giganotosaurus 02??? 000?? 0?00? 11?10 00010 01101 ???10 ?1110 10002 1110? ????? ??202 1211? ?1121 1??0? ?1?00 020?1 10121 ???00 100?0 01121 1?1?? ????1 11??2 01010 ????? 1?111 1110? 00200 00??0 00100 11012 ?01?0 ??111 001?0 1?1?0 0?100 ?1100 1?21? 2???? ?2?00 000?? ?1??0 ?10?? 0???0 1?0?? ????? ????? ????? ????? ????? ????? 00?11 01001 02111 10110 20120 1?120 02011 11000 22011 101?? 1?0?? ?1001 ?3122 1010? 10??? ????? ????? ????? ???0

Irritator ????? 1???? ?2?0? ????? ?0110 0200? 10001 0??04 00010 ?2011 10010 000?? 00011 ?1100 0???? 0?0?? ?11?1 0??10 ?1001 00010 21000 ?101? 10??? ??0?? ????? ????? 10?11 ?0??? 1101? 11??? ???1? ????? 1???? ????? ????? ????? ????? ????? ????? ????? ????? ????

Leshansaurus ????? ????? ?1?1? 1?001 ??1?0 [01]???1 ????? ????? ????? ????? ????? ????? ????? ?1000 0???? ????? ????0 00000 01002 1001? ?00?? ??0?? ????? ????? ????? ????? ????? ??00 ???0? ????? ??[01]0? 1100? 1?001 0??01 10000 121?0 000?? 0???? 00000 1100? ????? ????? ????? ????? ????? ????? ????? ????? ????? 1???? ????? ???0? 0???? ??011 0?011 1011? [12]0??? ????? 0??0? ????? [01]???? 10000 1???1 ?10?? ?2111 10?0? ????? ????? ????? ??0?? ????

Lourinhanosaurus ????? 1100? ????? ???0? ????? 1?1?0 00?00 0?0?0 0???1 ?1?0? ?1100 000?? ???0? 111?? ????? ????? ????? ????? ????? ????? ????? ????? 00?01 010?1 01111 1011? 2???? ?1??? 0?001 100?? 11011 101?0 110?? ???01 ?21?2 101?? ????? ????? ????? ????? ????

Magnosaurus ????? ????? ????? ????? ????? ????? ???0? ????? ????? ?????
????? ????? ????? ????? ????? ????? ????? ????? ????? ????? ????? ?????
????? ????1 10?11 ????? ????? ??000 0000? 0???? ????0 ???0? ????? ?????
????? ????? ????? ????0 ????? ????? ???00 ????? ????? ????? ????? ?????
????? ????? ????? ????? ????? ????? ?0??? 01??1 ????? ????? ????? ??0??
0???? ????? 1???? ???0? 110?0 01000 ?21?? ???0? 11??? ????? ????? ?????
????

Majungasaurus 02101 00010 00000 10000 00010 01000 00111 20?10 02000 0?101
20000 00202 02012 ?1111 01101 10000 10001 0100? 00000 010?0 1101? 10000
00010 10110 01010 01100 10001 00101 00000 00100 00100 01102 10011 00100
11000 12110 00000 10100 00001 021?1 11100 1201? 01000 011?1 011?1 0?011
10200 0010? ????? 0???? ????? ???0? ?0011 10000 00011 11111 ????? ?0???
1???? ????? 0???? 10011 0?100 01010 02102 11111 11102 1111? 0001? ?1101
???0

Mapusaurus ????? ?0??? 00000 11110 00010 01101 ?1010 11110 10002 1110?
?1111 102?2 12112 1???? ????[02] 01?00 0?0?? ????? ????? ????? ????? ?????
????? ??002 01010 ?0100 1???? ??100 00200 00?0? ??100 11012 ???01 ???1?
?0?00 10??0 001?? 01?00 10201 2???? ?2100 00??? ????? ?1?11 012?0 ????1
???10 ?10?? ????? 0???? 1???? ????0 00??? 0?0?1 02?11 10110 [12]???? ?????
0?011 ?1000 22??1 10111 1?00? 1100? ?3122 10101 10??2 11101 2101? ?0010
????

Marshosaurus 000?? 00000 00010 01000 11020 11001 ?0000 10?00 0???? ?????
?0?10 01?10 000?? ??100 0000? 10?0? 01?01 00020 00000 1001? 0?00? 1?0??
????? ????1 0001? ?0??? 1?011 ??001 00100 01000 00100 11001 ?0111 11000
10000 12110 00000 00120 01101 ?1??? ????? ????? ?10?? ????? 0?1?? ?????
????? 1???? ????? ????? ????? ????? 00?11 01001 01011 ?0110 01020 1?000
0?001 11000 ????? 1???? ????? ????? ????2 1???? ???0? ????? ????? ?????
??0?

Masiakasaurus 0???? 000?? 00100 10000 01000 01000 ???0? ????? ???10 ?????
????? ?0??2 0101? 10??? ????? ?0?00 1??00 ????0 00?00 01??0 0???0 100??
????? ??111 01000 01190 ??00? 00010 00000 00??? 00200 01102 ?0011 00100
11000 111?0 00000 10100 00000 02011 11000 1011? 01000 01?01 01111 10011
10100 001?? ????? ????? ????? ???00 1001? 10000 00011 ?1111 11120 10000
10010 01??1 00110 10011 02100 01010 02102 ?1111 11202 1111? 0011? ?1101
??00

Megalosaurus ????? ????0 000?0 11011 00?00 110?1 ?000? ????? ????? ?????
?1?11 1???? ????? ????? ????? ????? ????? ????? ????? ????? ????? ?????
????? ????0 00101 ????? 0?011 00001 0010? 01??0 0?100 ???01 ????? ???0?
????? 1???? ?0?1? ???10 00201 11000 01?00 00??? 010?? ???01 110?0 00001
21211 0?000 01000 0???? ????? ????? 00101 01011 01011 10113 ?00[12]? ?????
0?10? ?1?00 11101 10000 1?000 01000 0221? ?0?0? 1???? 1???? 1001? ?00??
????

Metriacanthosaurus ????? ????? ????? ????? ????? ????? ????? ????? ?????
????? ????? ????? ????? ????? ????? ????? ????? ????? ????? ????? ?????
????? ????? ????? ????? ????? ????? ????? ????? ????? ????? ?1001 ?????
????0 ??0?? 1?1?0 0011? 00?00 01211 1???? ?1?00 0?0?? ?1??? ?1??? ?????
????? ????? ????? ????? ????? ????? ????? 00??1 01001 01111 ?011? ?012?
1?0?0 0?101 ?01?1 11??1 10?11 11000 1??0? ??21? ????? ????? ????? ?????
????? ????

Monolophosaurus 001?1 000?0 0100? ????? 00000 2000? 10001 11013 00100 ?9001
2[01]110 00110 010?1 11100 01?02 10000 00??1 00010 00000 10??? ??0?? ??01?
1???? ??001 00001 00201 00?0? ??00? 0010? 0??00 00101 11001 0?001 00100
10?00 10110 00000 0?100 00101 ?10?0 ?1?00 0?00? 010?? ????? ????? ?????
????? ????? ????? ????? ????? ????? 00110 00101 01011 1011? 00?[12]0 100??
?0001 000?0 ????? ????? ????? ????? ????? ????? ????? ????? ????? ?????
??10

MSNM_V4047 112?1 20101 12101 20?00 00000 020?? 1??01 000?0 ????? ?????
????? ????? ????? ????? ????? ????? ????? ????? ????? ????? ????? ?????
????? ????? ????? ????? ????? ??010 1101? 10013 1111? ????? ????? ?????
????? ????? ????? ????? ????? ????? ????? ????? ????? ????? ????? ?????
????? ????? ????? ????? ????? ????? ????? ????? ????? ????? ????? ?????
????? ????? ????? ????? ????? ????? ????? ????? ????? ????? ????? ?????
?201

Neovenator 00??? 00000 01000 10101 00000 21001 ???10 11011 0???? ?????
????? ????? ????? ????? ????? ????? ????? ????? ????? ????? ????? ????1
????? ????1 10000 ????? ????? ??100 00100 01?02 00100 11012 ?1??? ??111
10100 12110 00100 01101 01011 0?0?? ?2100 001?? ?1?10 ?10?1 011?0 120??
????? ????? ????? ????? ????? ????? 01?11 01001 02211 10110 20120 1?120
00001 ?1011 020?1 10111 10001 11001 13122 1010? 10??? 1???? 21011 1?010
??00

Ornitholestes 000?? 000?0 0000? ????? 00000 2100? 1?000 01000 00101 0?001
2?110 00001 000?1 ??100 01?02 0?00? ?0??1 000?0 0???0 ????0 ??0?? ?????
????? ??000 00?0? ?0?0? 0?010 0111? 00000 0??00 00100 1100[12] ????? ????0
10010 1?0?0 00?00 01000 00001 0100? ?1?00 010?? 010?? 11??? ????? ??001
11100 00000 0???0 1??11 ??111 ??1?? 00?00 01011 12111 10110 20??? ?????
01001 00000 21??2 11??0 10000 1???? ????? ?0?0? ????? ????? 2001? ?0???
??00

Oxalaia 11??? ??1?1 1???? ????? ????? ????? ????? ????? ????? ????? ?????
????? ????? ????? ????? ????? ????? ????? ????? ????? ????? ????? ?????
????? ????? ????? ????? ??010 ???1? ??013 11??? ????? ????? ????? ?????
????? ????? ????? ????? ????? ????? ????? ????? ????? ????? ????? ?????
????? ????? ????? ????? ????? ????? ????? ????? ????? ????? ????? ?????
????? ????? ????? ????? ????? ????? ????? ????? ????? ????? ????? ?101

Piatnitzkysaurus ????? ????? 000?0 10000 10020 110?1 ???0? ????? ?????
????? ????? ????? ????? ?1??? 0???? ????? ????1 0?010 00000 10?11 01000

?10?? ????? ????2 010?1 ????? ????? ??001 0010? 01??? ??000 01001 ?0011
11000 10000 12110 00010 00120 0?101 11000 01?00 0?0?? 010?? ???01 011?1
12001 21110 1100? ?100? 1???? ????? ????? 001?1 01001 01011 ?011? 0002?
10000 00001 10000 11?11 10000 10000 01000 02212 1010? 10??? 1???? 1001?
?0??? ????

Piveteausaurus ????? ????? ????? ????? ????? ????? ????? ????? ????? ?????
????? ????? ????0 11?00 0???? ????? ????1 000?0 ?1002 100?? 1002? 110??
????? ????? ????? ????? ????? ????? ????? ????? ????? ????? ????? ?????
????? ????? ????? ????? ????? ????? ????? ????? ????? ????? ????? ?????
????? ????? ????? ????? ????? ????? ????? ????? ????? ????? ????? ?????
????? ????? ????? ????? ????? ????? ????? ????? ????? ????? ????? ?????
????

Proceratosaurus 000?? 000?0 0100? ????? 00000 2100? 1??0? ????? ??0?? ?????
20110 0000? ????? ????? ?1??2 0??0? ?0??? ????? ????? ????? ??0?? ?????
????? ??00[02] 00?0? ?0??? 00??1 ?1?11 0000? 0??00 00000 ????? ????? ?????
????? ????? ????? ????? ????? ????? ????? ????? ????? ????? ????? ?????
????? ????? ????? ????? ????? ????? ????? ????? ????? ????? ????? ?????
????? ????? ????? ????? ????? ????? ????? ????? ????? ????? ????? ?????
??00

Saurophaganax ????? ????? ????? ????? ????? ????? ????? ????? ????? ?????
????? ??0?? ????? ????? ????? ????? 0???? ????? ????? ????? ????? ?????
????? ????? ????? ????? ????? ????? ????? ????? ????? 1100? 1???? ?????
?0?00 1?1?0 00?0? ?0??? ???11 ????? ???00 ??0?? ????? ?10?? ????? ???01
2?010 1100? ????? ????? ????? ???00 001?1 01001 0???? ????? 20?[12]0 1?1?0
0??01 ????? 210?1 10??0 1???0 11001 ?21?? ????? ????? ????? ?00?? ?????
????

Shaochilong ????? ????? 000?? 11?10 10010 01101 11?00 ???1? ????? ?????
????? ????? ????? ?1?01 1???? ?0?00 02001 1???1 ?0000 100?0 011?? 120??
????? ????? ????? ????? ????? ??1?? ????? ????? ????? ????? ????? ?????

????? ????? ????? ????? ????? ????? ????? ????? ????? ????? ????? ?????
????? ????? ????? ????? ????? ????? ????? ????? ????? ????? ????? ?????
????? ????? ????? ????? ????? ????? ????? ????? ????? ????? ????? ?????
????

Shidaisaurus ????? ????? ????? ????? ????? ????? ????? ????? ????? ?????
????? ????? ???0? ?1100 0???? ????? ????1 10?0? ????? ????0 ????? ?????
????? ????? ????? ????? ????? ????? 0??0? 00??? ????? ???0? ?0110 000??
????? 1???? 001?0 0???? 01211 01000 01?00 ????? ??0?? ????? ????? ?????
????? ????? ????? ????? ????? ????? 00??0 ?1??1 ???11 10110 20?[12]0 ??00?
?20?? 0??0? ????? ????? ????? ????? ????? ????? ????? ????? ????? ?????
????

Siamotyrannus ????? ????? ????? ????? ????? ????? ????? ????? ????? ?????
????? ????? ????? ????? ????? ????? ????? ????? ????? ????? ????? ?????
????? ????? ????? ????? ????? ????? ????? ????? ????? ???0[12] ????? ?????
????? ????? ????? ????0 ???11 01000 ?0?00 0???? 010?? ????? ????? ?????
????? ????? ????? ????? ????? ????? 00201 01001 02?11 10112 20121 10000
??101 101?? ????? ????? ????? ????? ????? ????? ????? ????? ????? ?????
????

Sinraptor_dongi 00001 00000 00000 00000 01000 21111 11000 11010 00002 11000
01110 00100 01001 ?1100 00112 00100 02001 10110 00100 10010 01120 10011
1?111 11000 00011 00200 11111 00000 0010? 0?100 00100 11001 10110 00100
10000 12110 00110 00110 01211 010?0 ?1?00 0?000 010?? ?1?01 01110 0????
????? ????? ????? ????2? ????? ?11?0 00201 01001 01011 10112 20121 11000
02101 10101 11011 10111 10000 11000 02212 10101 10102 11101 21011 10010
1?00

Sinraptor_hepingensis 00001 00000 0000? ????? 01000 2111? 1?000 11011 00002
11000 01110 00100 01001 ?1100 00112 00100 0???1 1?120 00?00 ????? 01???
???1? 1???? ??000 0001? 00?00 1???1 ?000? 00?00 0?100 00100 11001 10110
00100 10000 12110 00110 001?0 01211 ?1100 01100 0100? 0101? 11001 01110

0?0?? ????? ????? ????? ????? ????? ????? 00001 01001 01?11 10112 [12]0021 11010 02101 10?10 110?1 10111 1?00? ????? ????? ????? ????? ????? ????? ????? ??0?

Suchomimus 11110 10101 12101 00?00 00010 0200? 1??0? ??0?0 ????? ????? ????? ????? ????? ?1??? ????? ???11 0110? ????? ????? ????? ????? ????? ????? ????1 10??? ???1? ????? ??010 01011 0?013 00101 1100[12] ????? ??100 ?0001 121?1 11010 00110 00201 ?10?0 ???00 000?? ?1001 ?0001 011?0 10001 21111 00010 01101 0???? ????? ???10 0011? 010?1 00011 10110 00?[12]? 0?00? 00001 1?00? 11??1 10100 11010 0100? ?4121 1010? 10202 1??0? 1???? ????? ?010

Torvosaurus 00??? 000?0 010?? 11011 00010 12000 ??00? ???00 ?1012 00001 ?0111 11011 000?[01] ????? ????? 00111 1?01? ????? ????? ????? ????? ????? ????? ????0 10?0? ????? ????? ??000 00100 01001 00100 11011 01021 00100 10001 12110 00011 00110 00101 11000 01110 000?? 010?1 01001 11000 00001 21211 00000 0100? 0[12]??? 00110 11010 00001 01011 01011 10113 000[12]? 10000 02001 01000 1110? 10000 11000 01000 02121 10101 10102 1110? 1001? ?0??? ??00

Tyrannotitan ????? ????? ????? ????? ????? ????? ???1? ????? ????? ????? ?1111 ????? ????? ????? ????? ????? ????? ????? ????? ????? ????? ????? ????? ????2 0?010 ?0??? ????? ??1?? 0?20? 0???? ????0 1101? 1???? ???1? ??100 1?1?0 0??00 ?1?00 1?2?1 ????? ??000 ??0?? ????? ???11 ????0 1???? ????? ????? ????? ????? ????? ????? ????? ????? ????? ?0??? ?0?20 1???? ?20?? ???0? 220?1 ?0111 1?0?0 1???? ???2? ????? ????? ????? ????? ??010 ????

Yangchuanosaurus_magnus 0000? 00000 0000? ????? 00000 2111? 1?000 11011 00002 ?1000 01110 0010? 010?1 ?1??0 ?0?12 0???0 ?0??? ????? ????? ????? ????? ??011 1???1 ??000 00?1? 00??? 1???1 ?0??? 00?0? 0??00 00100 1100[12] ?0110 001?0 10000 12110 00100 0?1?0 00111 01000 0?000 0100? 010?? ????? ????? ????? ????? ????? ????? ????? ????? ????? 00000 01001 01?11 10110

10120 ?100? 02001 1?111 11??0 101?1 10??? ?100? ?211? ?0101 10102 1??0? ????? ????? ??0?

Yangchuanosaurus_zigongensis ????? 00?0? ?1??? ??00? 1100[12] ????? 0010? ?0000 12110 00100 0?1?0 0?011 ?1??0 ??000 ?100? ????? ???01 011?0 ???01 10210 01000 01000 1??2? 00111 111?? 00001 010?1 0??11 10110 101[12]0 ?100? 02001 10111 11?1? ?0??1 10?00 0?00? ?211[12] 10?0? ?010? ????? ????? ????? ????

References

1. Carrano MT, Benson RBJ, Sampson SD. The phylogeny of Tetanurae (Dinosauria: Theropoda). J Syst Palaeontol. 2012;10(2):211-300. doi: dx.doi.org/10.1080/14772019.2011.630927.
2. Carrano MT, Sampson SD. The Phylogeny of Ceratosauria (Dinosauria: Theropoda). J Syst Palaeontol. 2008;6(2):183–236. doi: 10.1017/S1477201907002246.
